# Analysis of Research Progress on the Chemical Constituents and Pharmacological Activities of Er-Shiwei Roudoukou Wan

**DOI:** 10.3390/ph19010052

**Published:** 2025-12-25

**Authors:** Kai Hao, Lingxiao Chen, Zongyao Wu, Cizhen Danzeng, Xiaorui Cheng

**Affiliations:** 1 Innovative Institute of Chinese Medicine and Pharmacy, Shandong University of Traditional Chinese Medicine, Jinan 250355, China; 15129836766@163.com (K.H.); wsclx1@126.com (L.C.); 2 The Key Laboratory of Tibetan Medicine and Plateau Biology, Xizang University of Tibetan Medicine, Lhasa 850010, China; 3 Department of Tibetan Medicine, Xizang University of Tibetan Medicine, Lhasa 850010, China

**Keywords:** Ershiwei Roudoukou Wan, Tibetan medicine, tranquillization, bibliometrics, chemical constituents, pharmacological effects

## Abstract

Ershiwei Roudoukou Wan, a traditional Tibetan medicine, is known for its sedative and tranquilizing properties. Although considerable progress has been made in characterizing its chemical constituents and pharmacological mechanisms, a comprehensive and systematic evaluation remains limited. This study integrates bibliometric analysis with a systematic literature review to summarize current research trends, clarify the chemical basis, and assess pharmacological evidence, thereby supporting rational clinical use. Publications on Ershiwei Roudoukou Wan and its component herbs were retrieved from the Web of Science database. After screening, 7869 articles were analyzed using CiteSpace to generate knowledge maps. Research hotspots centered on pharmacological activities, chemical composition, and methodological advances. Clinically, the formula has shown efficacy in conditions including heart–gallbladder syndrome, “Ninglong disease” and gynecological disorders. Analytical methods for several marker compounds have been established, and individual herbs contain diverse bioactive constituents—predominantly terpenoids, flavonoids, polysaccharides, and tannins. Pharmacological investigations highlight cardiovascular protection, immunomodulatory and anti-inflammatory effects, anticancer activity, and neuroprotection. Despite these advances, experimental studies on the complete formulation and large-scale clinical trials remain scarce. Future research should leverage advanced analytical and pharmacological techniques to elucidate the integrated mechanisms of action and promote the modernization of Tibetan medicine.

## 1. Introduction

Tibetan medicine is prominent in China’s national medical system, distinguished by its deep historical roots and unique therapeutic philosophies. Among its classic formulations, Ershiwei Roudoukou Wan (RDK20) stands out as a traditional prescription that received official approval for clinical use on 6 September 2002 (National Drug Approval Number Z20023260). Designed to calm the mind and alleviate agitation, this remedy is primarily indicated for symptoms associated with “Ninglong disease”—such as irritability, confusion, insomnia, dizziness, memory impairment, tinnitus, tremors, and heart palpitations [1]. The formula contains 20 medicinal substances, encompassing botanical, animal, and mineral sources. Key plant-derived components include *Myristica fragrans* Houtt (Roudoukou, RDK), *Dalbergia odorifera* (Jiangxiang, JX), *Aquilaria sinensis* (Chenxiang, CX), *Choerospondias axillaris* (Guangzao, GZ), *Carthamus tinctorius* (Honghua, HH), *Carum carvi* (Zanghuixiang, ZHX), *Syzygium aromaticum* (Dingxiang, DX), *Allium sativum* (Dasuan, DS), *Amomum kravanh* (Doukou, DK), *Ferula sinkiangensis* (Awei, AW), *Amomum tsao-ko* (Caoguo, CG), *Terminalia chebula* Retz. (Hezi, HZ), *Boswellia carterii* (Ruxiang, RX), *Terminalia belerica* Roxb. (Maohezi, MHZ), *Senegalia catechu* (Ercha, EC), *Phyllanthus emblica* (Yuganzi, YGZ), Rhizoma ex Radlx Bergeniae (Ligadu, LGD), and *Santalum album* (Tanxiang, TX) [2]. The formula includes *Calculus Bovis* (Niuhuang, NH), derived from animal sources, and *Calx Pulveratum* (Shihuihua, SHH) as a mineral component, enhancing its broad therapeutic efficacy.

RDK20 has received limited attention in contemporary basic research despite its long-standing use in clinical practice. This situation is mainly attributable to the relatively late initiation of efforts to modernize Tibetan medicine, resulting in a paucity of systematic investigations into its chemical composition and pharmacological properties. A bibliometric analysis was conducted on RDK20 and its constituent herbs to systematically sort out the research status of RDK20 and identify research gaps, based on literature indexed in the Web of Science database from 2005 to 2025 [3]. Using CiteSpace, a visual knowledge map was generated to identify key research domains and track the evolution of this field over time. This review further offers a comprehensive overview of existing research on RDK20, encompassing its clinical applications, chemical characterization, and pharmacological activities, to facilitate its standardized and evidence-based use in practice.

## 2. Overview of Ershiwei Roudoukou Wan-Related Research Based on Bibliometric Analysis

**Figure 1:**
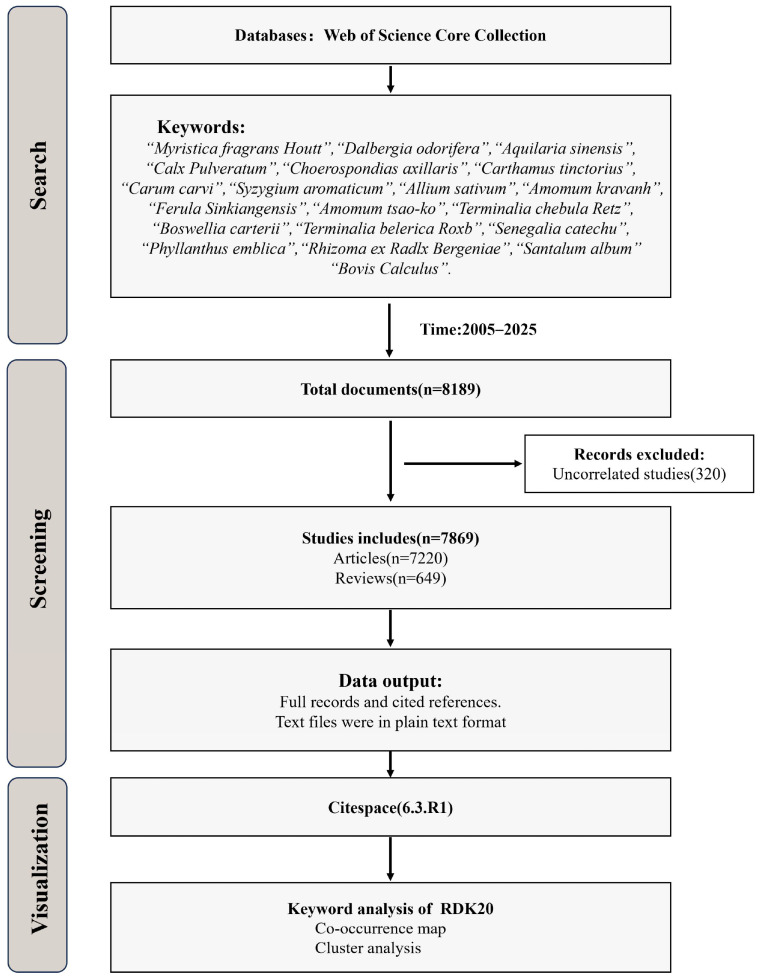
Bibliometric Research Workflow.

To gain a comprehensive understanding of the research landscape surrounding RDK20, a search was conducted in the Web of Science database for articles published from 1 January 2005 to 1 December 2025. Given the limited number of studies directly focused on RDK20, the search scope was expanded to include its individual constituents. Since the therapeutic efficacy of multi-component Tibetan medicine formulas like RDK20 relies on the synergistic effects of their components, and studies on individual ingredients can indirectly reflect the formula’s research progress. The search encompassed 18 plant-derived ingredients—such as *Myristica fragrans* Houtt., *Dalbergia odorifera*, *Aquilaria sinensis*, *Carthamus tinctorius*, and *Santalum album*—as well as animal- and mineral-based substances, including artificial bezoar and calcite. This approach yielded a total of 8189 records. After removing duplicates and irrelevant entries, 7869 studies were retained for further analysis. The complete records and citation data were exported in plain text format and analyzed using CiteSpace 6.3.R1 to construct a visual knowledge map. The detailed search strategy is presented in Figure 1.

**Figure 2:**
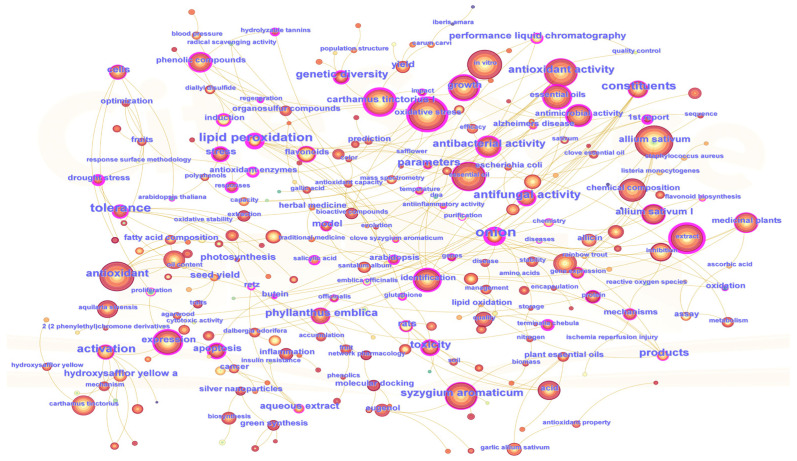
Keyword co-occurrence analysis. The dimensions of each node correspond to the frequency of keyword appearances; different colors represent keywords appearing in different years.

A keyword co-occurrence analysis pertaining to RDK20 was performed using CiteSpace, based on publications retrieved from the Web of Science database. As illustrated in Figure 2, the resulting network comprises 263 nodes and 282 links, yielding a density of 0.008. Although the network is sparse, the structure reveals meaningful associations among research themes. The analysis indicates that current research on RDK20 primarily concentrates on three thematic areas: pharmacological effects, chemical composition, and research methodologies, with pharmacological effects emerging as the central theme. Frequently occurring keywords—such as oxidative stress (552), antioxidant (484), antibacterial activity (366), apoptosis (203), inflammation (99), and Alzheimer’s disease (21)—underscore significant interest in the formula’s anti-inflammatory, antioxidant, and antimicrobial properties, as well as its effects on programmed cell death. In the domain of chemical composition, research predominantly focuses on identifying bioactive compounds contributing to the therapeutic efficacy of RDK20. High-frequency terms—such as volatile oil (488), chemical composition (380), phenolic compounds (194), flavonoids (164), eugenol (108), and hydroxysafflor yellow A (99)—reflect sustained efforts to characterize principal constituents and elucidate their chemical structures. Regarding analytical methodologies, keywords such as extraction (471), identification (362), and high-performance liquid chromatography (72) indicate consistent emphasis on analytical precision and quality control. Such emphasis is particularly critical for multi-component traditional formulations like RDK20, in which the safety and consistency of clinical application depend on the accurate characterization of their constituents. Moreover, several individual herbs—such as safflower (*Carthamus tinctorius*), garlic (*Allium sativum*), clove (*Syzygium aromaticum*), and agarwood (*Aquilaria sinensis*)—have garnered comparatively greater research attention. Studies on these ingredients have yielded important insights that contribute to elucidating the broader pharmacological profile of the formula as a whole.

**Figure 3:**
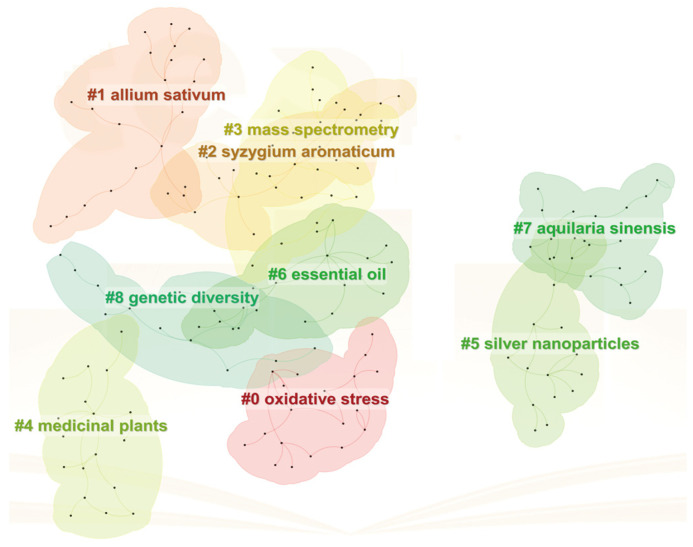
Keyword Cluster Analysis.

A cluster analysis was performed using CiteSpace to group keywords with similar thematic content, resulting in a visual map that delineates the structural landscape of RDK20 research (Figure 3). The analysis identified nine primary clusters, numbered from #0 to #8. The overall clustering quality was robust, as evidenced by a modularity score (Q) of 0.8606—well above the conventional threshold of 0.3—indicating a clear demarcation between thematic areas. An average silhouette score (S) of 0.9054 further reflects high internal consistency and reliable cluster homogeneity. Lower-numbered clusters generally encompassed a larger set of keywords. Several clusters were particularly prominent, notably those associated with oxidative stress, garlic, clove, mass spectrometry, medicinal plants, and volatile oils. These findings indicate that current research continues to emphasize the pharmacological mechanisms of action and the functional roles of individual medicinal components within the formula.

**Figure 4:**
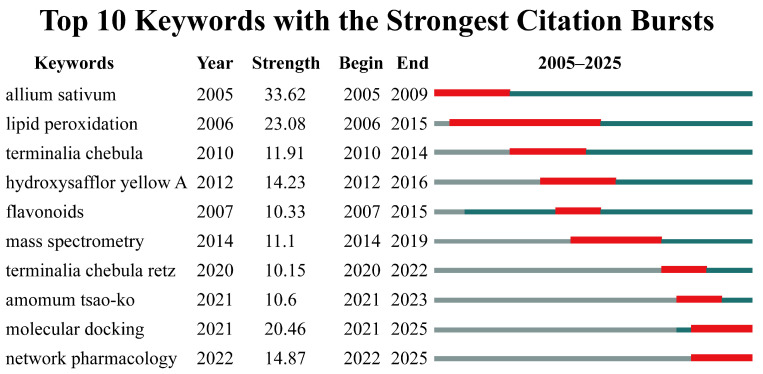
Keyword Burst Analysis. The red horizontal bars highlight the specific time periods when each keyword experienced a significant increase in citation frequency, with the length of the bar representing the burst duration.

A keyword burst analysis was performed to elucidate the temporal evolution of RDK20-related research. Figure 4 highlights the top 10 keywords exhibiting the most prominent citation bursts and illustrates temporal shifts in keyword bursts across different periods. Between 2005 and 2012, the most pronounced bursts were associated with single herbs and their antioxidant activities, signaling an initial phase of chemical profiling. For instance, interest in garlic emerged as early as 2005, followed by lipid peroxidation (2006), *Terminalia chebula* (2010), and hydroxysafflor yellow A (2012). More recently, the emergence of terms such as molecular docking and network pharmacology in 2022 indicates an increasing emphasis on mechanistic investigations and systems-level methodologies—aligning with a broader trend in Tibetan medicine research toward integrating traditional formulations with modern computational pharmacology to validate their therapeutic mechanisms.

The duration of these citation bursts varied considerably. Lipid peroxidation sustained research attention for nine consecutive years (2006–2015), whereas mass spectrometry remained active for five years. Such prolonged bursts reflect sustained interest in oxidative stress-related pathways and analytical methodologies in the context of traditional medicine research. In terms of burst intensity, garlic, lipid peroxidation, and molecular docking exhibited the highest strength. Garlic, widely recognized for its culinary and medicinal applications, continues to attract considerable research interest due to its diverse pharmacological activities. The persistent prominence of lipid peroxidation underscores the central role of antioxidant mechanisms in disease prevention and therapeutic intervention.

The recent emergence of molecular docking reflects a methodological shift toward computational tools for elucidating molecular interactions within complex herbal formulations. Such technologies provide valuable insights into potential synergistic effects and support the integration of traditional formulations like RDK20 into contemporary drug discovery frameworks.

## 3. Clinical Application

Clinical evidence regarding Ershiwei Roudoukou Wan is primarily derived from observational studies, retrospective analyses, and clinical experience reports within Tibetan medicine practice. To date, large-scale randomized controlled trials remain limited. Therefore, the following section summarizes reported human-based clinical outcomes, with an emphasis on therapeutic indications, observed efficacy, and overall trends rather than mechanistic interpretation.

### 3.1. Cholecardia Syndrome

Cholecardia syndrome, also referred to as gallbladder–heart syndrome, is characterized by secondary cardiac dysfunction associated with gallbladder or biliary tract disorders. Clinically, this condition may manifest as arrhythmia, myocardial injury, acute coronary syndrome, or heart failure, and is thought to involve neurogenic reflexes, bile acid dysregulation, and metabolic disturbances.

In a clinical observational study involving 132 patients diagnosed with cholecardia syndrome, participants were divided into two groups. The control group received a conventional Tibetan medicinal regimen, while the treatment group was administered a combination therapy that included Ershiwei Roudoukou Wan. The overall response rate in the treatment group reached 92.42%, which was significantly higher than that of the control group (77.27%, *p* < 0.05) [4]. These findings suggest that formulations containing Ershiwei Roudoukou Wan may provide additional clinical benefit when incorporated into combination therapy for cardiovascular–biliary disorders.

Although these results are encouraging, it should be noted that the study design was observational, and standardized outcome measures were limited. Nevertheless, the reported clinical improvement supports the continued use of Ershiwei Roudoukou Wan as part of integrative Tibetan therapeutic strategies for cholecardia syndrome.

### 3.2. Ninglong Syndrome

In Tibetan medicine, Ninglong-related disorders are commonly understood as functional cardiovascular conditions associated with emotional dysregulation, dietary imbalance, and disturbances in qi and blood circulation. Clinically, patients may present with palpitations, chest tightness, insomnia, dizziness, memory impairment, and emotional instability [2]. These symptom clusters show considerable overlap with what is classified in modern medicine as cardiac neurosis or stress-related cardiovascular dysfunction.

Retrospective analyses of Tibetan medical prescriptions indicate that Ershiwei Roudoukou Wan is frequently employed in the management of Ninglong-related conditions. An analysis of 80 prescriptions issued by a senior Tibetan physician over a 25-year period identified Ershiwei Roudoukou Wan in 17 cases, reflecting its recurrent clinical application in this context [5]. Traditionally, the formula is valued for its calming and stabilizing effects, particularly in patients exhibiting agitation, restlessness, or disordered speech.

Further clinical support is provided by a study involving 123 patients diagnosed with neurosis (referred to as Soron disease in Tibetan medicine), who received treatment regimens incorporating Ershiwei Roudoukou Wan in combination with other Tibetan formulations. After treatment, an overall effectiveness rate of 95% was reported, with most patients exhibiting marked symptom improvement or recovery [6]. Although these findings are derived from non-randomized clinical observations, they collectively support the relevance of Ershiwei Roudoukou Wan in managing functional cardiovascular and neuropsychological disorders.

### 3.3. Gynecological Conditions

Ershiwei Roudoukou Wan has also been applied in the clinical management of gynecological conditions, particularly those associated with hormonal fluctuations and autonomic dysregulation. In Tibetan medical theory, such conditions are often attributed to imbalances in kidney function, liver qi stagnation, and disrupted circulation of qi and blood [7].

In a clinical observational study of 60 patients with perimenopausal syndrome, treatment with Ershiwei Roudoukou Wan combined with psychological intervention resulted in a reported effectiveness rate of 98.33%, with improvements observed in symptoms such as hot flashes, palpitations, and sleep disturbances [8]. The treatment was generally well tolerated, and no significant adverse reactions were reported.

Additional evidence comes from a study involving 40 women with menstrual-related headaches who received Tibetan medicinal regimens containing Ershiwei Roudoukou Wan alongside other formulations. Clinical outcomes indicated an overall effectiveness rate of 97.50%, with the majority of patients experiencing complete or partial symptom relief [9]. Although these studies vary in design and outcome assessment, they consistently suggest a potential role for Ershiwei Roudoukou Wan in alleviating gynecological symptoms linked to emotional and systemic imbalance.

### 3.4. Other Applications

Beyond its traditional indications, Ershiwei Roudoukou Wan has been reported in recent clinical practice as a supportive therapy for neuropsychological symptoms associated with post-COVID conditions. Long COVID is characterized by persistent symptoms such as fatigue, cognitive impairment, mood disturbances, and sleep disorders lasting more than three months after acute infection [10].

Preliminary clinical observations suggest that Ershiwei Roudoukou Wan may alleviate symptoms such as anxiety, depression, and insomnia in this context, consistent with its traditional sedative and tranquilizing properties [11]. However, current evidence remains anecdotal or observational, and systematic clinical evaluation is still lacking.

### 3.5. Summary of Clinical Application

Overall, existing clinical evidence indicates that Ershiwei Roudoukou Wan has been applied in a range of conditions involving cardiovascular dysfunction, emotional dysregulation, and gynecological disorders. Most available data are derived from observational and retrospective studies, which support potential efficacy and safety but do not yet provide definitive conclusions. Further well-designed clinical trials are needed to substantiate these findings and to clarify the therapeutic role of Ershiwei Roudoukou Wan in modern clinical practice.

## 4. Chemical Composition

### 4.1. Chemical Constituents of the Whole Formula

Despite its long-standing clinical use, chemical investigations of Ershiwei Roudoukou Wan have so far focused predominantly on selected marker compounds rather than the integrated chemical profile of the complete formula. Quantitative analyses of compounds such as hydroxysafflor yellow A and dehydrodieugenol using RP-HPLC methods have provided reference points for quality control [12,13,14]. However, these isolated measurements do not adequately reflect the multi-layered chemical interactions that are characteristic of Tibetan compound prescriptions.

From a pharmacological perspective, this limitation is nontrivial. The therapeutic effects of Ershiwei Roudoukou Wan are unlikely to be driven by a single dominant constituent, but rather by the coordinated action of chemically distinct components. At present, the absence of holistic profiling data constrains any attempt to link chemical composition with integrated biological effects. Comprehensive analytical strategies, including UPLC-QTOF-MS-based profiling and metabolomic approaches, are therefore essential to move beyond descriptive chemistry toward mechanistically meaningful interpretation.

### 4.2. Chemical Constituents of the Single-Flavor Drugs

Based on volatility and physicochemical characteristics, the constituents identified from individual herbs can be broadly divided into non-volatile compounds and volatile (essential oil) components. This classification provides a functional framework for understanding how chemically diverse substances may collectively contribute to the overall pharmacological profile of the formula.

In the available literature, many constituents are reported as LC–MS/MS annotations, which are valuable for compositional profiling but do not always provide full structural certainty. When compounds are isolated and formally identified, confirmation is typically supported by 1D/2D NMR (most often ^1^H and ^13^C, and in some cases ^15^N or ^31^P for heteroatom-rich structures) [15]. For molecules with stereochemical complexity, the configuration of chiral centers may be further supported by single-crystal X-ray diffraction when suitable crystals are available [16]. For animal- and mineral-derived ingredients, elemental analysis can also be informative, as it helps verify the presence and proportion of heavy elements and complements spectrometric identification [17].

For a complete list of reported constituents and the corresponding sources, see Table S1 (Refs. [18,19,20,21,22,23,24,25,26,27,28,29,30,31,32,33,34,35,36,37,38,39,40,41,42,43,44,45,46,47,48,49,50,51,52,53,54,55,56,57,58,59,60,61,62,63,64,65,66,67,68,69,70,71,72,73,74,75,76,77,78,79,80,81,82,83,84,85,86,87,88,89,90,91,92,93,94,95,96,97,98,99,100,101,102,103,104,105,106,107,108,109,110,111,112,113,114,115,116]).

#### 4.2.1. Non-Volatile Constituents

##### Flavonoids

Flavonoids are widely distributed across several component herbs, including *Dalbergia odorifera*, *Aquilaria sinensis*, *Terminalia chebula*, *Phyllanthus emblica*, and *Carthamus tinctorius* (Table S1). They span multiple structural subclasses and are commonly linked to antioxidant and anti-inflammatory activities. While most evidence stems from single-herb studies, the recurrence and abundance of flavonoids suggest a key role in the cardiovascular and neuroregulatory actions ascribed to the full formula. Notably, variations observed in flavonoid profiles—such as those in *Aquilaria sinensis*—indicate that regional and botanical factors may influence efficacy [55,65,117].

##### Organic Acids

Organic acids represent another recurrent class of non-volatile constituents, particularly in fruit-derived components such as Choerospondias axillaris and *Phyllanthus emblica*. Compounds including gallic acid, malic acid, and quinic acid have been identified using LC–MS-based techniques [33,55]. Gallic acid, which serves as a quality control marker in the Chinese Pharmacopoeia (2020 edition), is shared by multiple ingredients within the formula.

From an interpretive standpoint, the widespread occurrence of organic acids suggests a potential role in redox regulation and metabolic modulation. While direct evidence linking these compounds to the clinical effects of Ershiwei Roudoukou Wan remains limited, their chemical properties and established bioactivities indicate that they may contribute to the formula’s antioxidant profile.

##### Tannins

Several components of Ershiwei Roudoukou Wan, notably Terminalia chebula, Phyllanthus emblica, and Senegalia catechu, are rich sources of tannins. Extensive phytochemical studies have identified structurally complex hydrolysable and condensed tannins, including chebulinic acid, corilagin, and catechin oligomers [57,118,119,120]. Given their strong protein-binding capacity and redox activity, tannins are frequently implicated in anti-inflammatory and antimicrobial effects. In the context of Ershiwei Roudoukou Wan, the co-existence of tannin-rich herbs suggests a cumulative contribution to these activities, although direct validation at the formula level has yet to be established.

Because tannins often occur as oligomers with closely related structures, MS-based profiling alone may be insufficient for unambiguous assignment. In Senegalia catechu, condensed tannins have been characterized by combining MALDI-TOF MS with ^1^H, ^13^C and ^31^P NMR, which helps resolve catechin/epicatechin oligomers and their linkage patterns [118].

##### Polysaccharides

Polysaccharides with diverse monosaccharide compositions and molecular weights have been isolated from multiple herbs in the formula, including Allium sativum, Phyllanthus emblica, Terminalia chebula, Carthamus tinctorius, and Boswellia [121,122,123,124,125,126,127,128,129]. These macromolecules are primarily associated with immunomodulatory and antioxidant activities, although their precise roles within the intact formula remain to be clarified. At present, polysaccharide-related studies remain largely confined to single-herb investigations. Nevertheless, their repeated occurrence across multiple components suggests that they may collectively support systemic regulation, particularly in immune and stress-related contexts.

#### 4.2.2. Volatile Constituents (Essential Oils)

Volatile components constitute another important chemical layer of Ershiwei Roudoukou Wan, contributing both to sensory characteristics and biological activity. Essential oil analyses using GC–MS have consistently identified terpenoids and phenylpropanoids as dominant constituents.

##### Terpenoids

Terpenoids, including monoterpenes and sesquiterpenes, are abundant in aromatic herbs such as *Myristica fragrans*, *Aquilaria sinensis*, *Boswellia*, and *Santalum album* [18,65,104,130,131,132,133,134,135]. These compounds are frequently associated with anti-inflammatory, antimicrobial, and central nervous system-related effects, which aligns with the traditional indications of the formula for calming and tranquilizing purposes.

##### Phenylpropanoids

Phenylpropanoids, although less abundant, add further structural and functional diversity. Compounds such as syringic acid, caffeic acid derivatives, and lignan-type phenylpropanoids have been identified in *Amomum kravanh* and Santalum album, expanding the known chemical spectrum of these herbs [18,104]. At present, studies on these macromolecular constituents remain largely confined to structural characterization and in vitro bioactivity assays. Their contribution to the therapeutic effects observed in animal models or clinical settings of the complete formula has yet to be systematically evaluated.

In addition, various hydrocarbons and oxygenated derivatives have been detected across multiple components, potentially influencing bioavailability and synergistic interactions among constituents.

#### 4.2.3. Summary and Compositional Features

**Figure 5:**
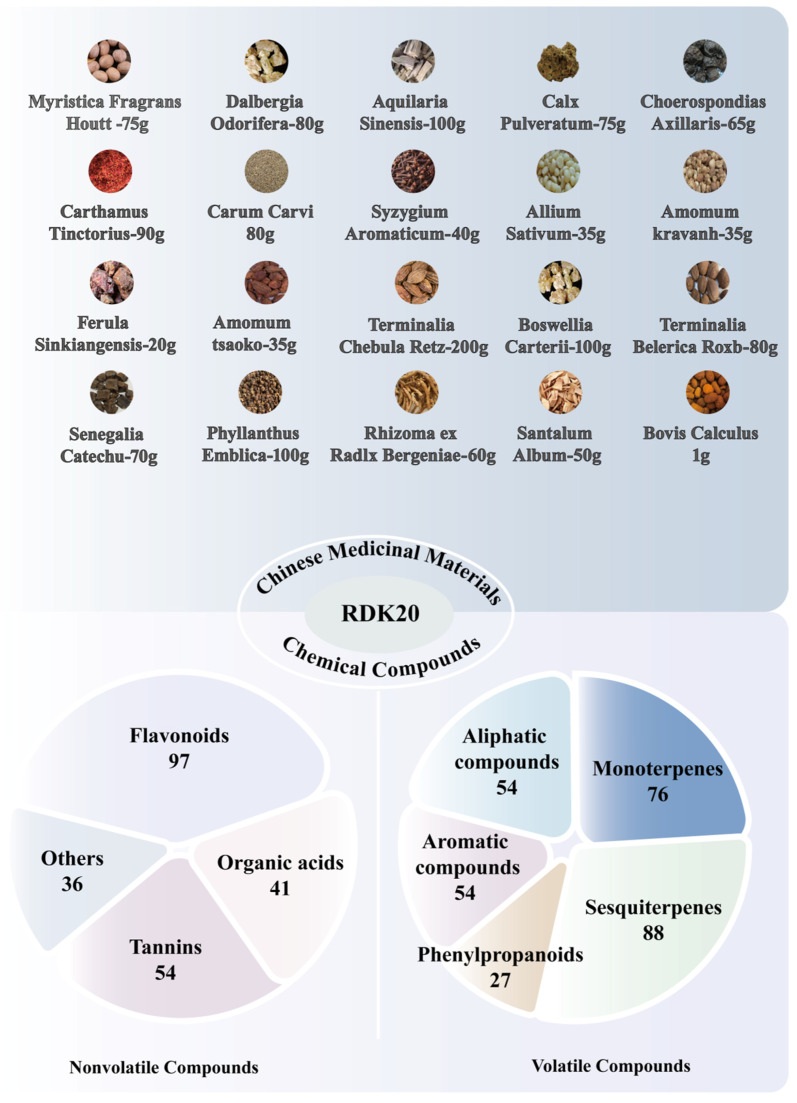
Herbal Composition and Chemical Constituents of Ershiwei Roudoukou Wan.

**Figure 6:**
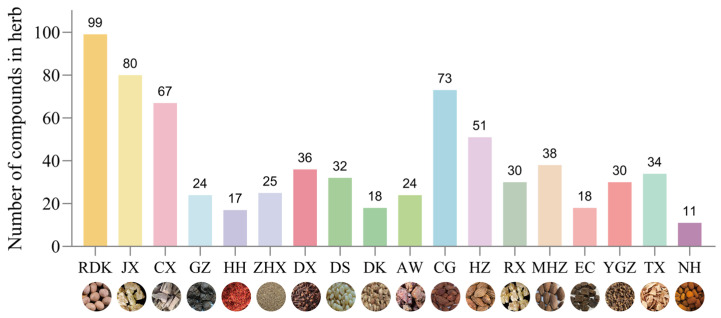
Number of Chemical Constituents Identified in Individual Herbs of Ershiwei Roudoukou Wan. This figure illustrates the number of chemical constituents identified in each individual medicinal ingredient of RDK20. The x-axis represents the abbreviations of the herbal names in pinyin (RDK for *Myristica fragrans* Houtt, JX for *Dalbergia odorifera*, CX for *Aquilaria sinensis*, GZ for *Choerospondias axillaris*, HH for *Carthamus tinctorius*, ZHX for *Carum carvi*, DX for *Syzygium aromaticum*, DS for *Allium sativum*, DK for *Amomum kravanh*, AW for *Ferula Sinkiangensis*, CG for *Amomum tsao-ko*, HZ for *Terminalia chebula* Retz, RX for *Boswellia carterii*, MHZ for *Terminalia bellirica* Roxb, EC for *Senegalia catechu*, YGZ for *Phyllanthus emblica*, TX for *Santalum album*, NH for Calculus Bovis). The y-axis indicates the number of reported chemical constituents in each herb.

The constituent herbs of Ershiwei Roudoukou Wan exhibit marked chemical diversity. Volatile constituents are primarily terpenoids and phenylpropanoids, whereas the non-volatile components largely include flavonoids, organic acids, tannins, and polysaccharides (Figure 5). Based on compound-level analysis (Figure 6), *Myristica fragrans* contributes the greatest number of distinct molecules, totaling 99. Altogether, 400 compounds have been identified across the 18 herbs, with 16 shared among four or more species—gallic acid, caffeic acid, and ferulic acid among them—indicating potential relevance to the formula’s overall bioactivity.

By category, terpenoids dominate, accounting for 147 compounds (36.8% of the total), underscoring their significance as a core chemical group in this traditional Tibetan formulation.

## 5. Pharmacological Activities

Existing studies suggest that RDK20 exhibits a broad range of biological activities, most frequently discussed in the context of inflammation, immune regulation, oxidative stress, tumor-related processes, and nervous system function. At the same time, research directly addressing the complete formula is still scarce, with much of the current knowledge extrapolated from individual herbs or isolated constituents. To clarify the strength and limitations of this evidence, the reported pharmacological findings are summarized and categorized in Table 1 according to study model and test material.

### 5.1. Cardiovascular Protection

Myocardial ischemia–reperfusion (MI/R) injury refers to the paradoxical worsening of myocardial damage upon the restoration of blood flow following ischemia, often transforming reversible injury into irreversible necrosis. Several constituents of Ershiwei Roudoukou Wan have demonstrated cardioprotective effects through distinct but convergent mechanisms.

Flavonoids from *Dalbergia odorifera* reduce serum levels of CK and ALT, along with oxidative stress markers such as nitric oxide and hydrogen peroxide, likely by improving cellular energy metabolism [136]. These findings underscore a role in attenuating myocardial injury via modulation of redox and metabolic pathways. Lipid metabolism—another key factor in cardiovascular disease—is targeted by multiple components. Methanolic extracts of *Choerospondias axillaris* exert lipid-lowering effects in vivo [132], while *Amomum* species enhance endothelial function, regulate energy metabolism, and diminish oxidative injury [137]. Clinically, Carthamus tinctorius has been shown to normalize lipid profiles and reduce myocardial injury biomarkers [138]. Together, these agents appear to act on vascular tone and lipid homeostasis, which are tightly linked to atherosclerosis progression.

*Terminalia bellirica* extract has been shown to reduce CK-MB levels and protect against cardiotoxicity induced by doxorubicin and isoproterenol. It also restores serum biochemical markers (ALT, AST, ALP, uric acid) and corrects lipid disturbances by reducing total cholesterol and triglycerides while increasing HDL cholesterol [139]. These broad-spectrum effects suggest potential utility in mitigating both ischemic and drug-induced cardiac injury.

**Figure 7:**
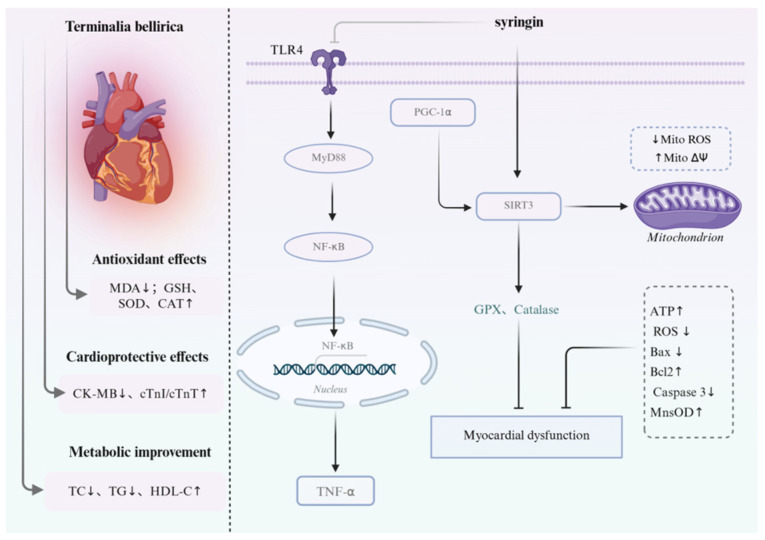
The protective mechanism of active ingredients in Ershiwei Roudoukou Wan on cardiac function. ↑ and ↓ indicate increase and decrease, respectively; solid arrows indicate activation/promotion; T-bar arrows indicate inhibition/blockade.

In diabetic cardiomyopathy models, syringin from *Syzygium aromaticum* improves cardiac function and suppresses BNP, CK-MB, and cardiac troponin I. Mechanistically, it inhibits the TLR4/MyD88/NF-κB pathway and downstream inflammatory mediators including NLRP3, IL-1β, IL-6, and TNF-α, while activating the PGC1α/SIRT3 axis to enhance mitochondrial function (Figure 7). Notably, eugenol—the major component of clove—augments these effects by stabilizing mitochondrial membrane potential and reducing mitochondrial ROS, reinforcing its antioxidant and anti-inflammatory role [140].

Taken together, these studies highlight a recurring pattern across different constituents: modulation of oxidative stress, lipid metabolism, inflammation, and mitochondrial integrity. Such converging mechanisms may underlie the synergistic cardioprotective potential of Ershiwei Roudoukou Wan.

### 5.2. Immunomodulatory and Anti-Inflammatory Activities

The immune system, composed of specialized organs, cells, and signaling molecules, plays a vital role in maintaining physiological balance through immune surveillance and regulation [141]. Beyond defending against pathogens, it contributes to delaying aging, stabilizing internal environments, and suppressing tumor development [142]. However, modern lifestyle factors—such as environmental pollutants, chronic stress, and physical inactivity—have disrupted immune homeostasis and contributed to the rising prevalence of immune-related disorders. Although biologics like cytokines and immunoglobulins have improved disease management, concerns over side effects and long-term safety highlight the need for safer immunotherapeutic alternatives.

Several constituents of Ershiwei Roudoukou Wan exhibit promising immunomodulatory effects. Garlic-derived polysaccharides (GPs), for example, promote NO, IL-6, and TNF-α production in RAW264.7 macrophages and induce morphological features of activation in vitro. In vivo, GPs reverse cyclophosphamide-induced immunosuppression by restoring serum cytokine and immunoglobulin levels and preserving immune organ structure. These effects are partially attributed to gut microbiota modulation and enhanced short-chain fatty acid production [143]. Likewise, a water-soluble polysaccharide isolated from Phyllanthus emblica stimulates splenocyte proliferation and exhibits antioxidant properties, suggesting further immune-supportive potential [144].

Uncontrolled inflammation—although integral to host defense—often underpins the development of chronic diseases such as arthritis, dermatitis, and gout. Multiple RDK20 components have shown anti-inflammatory efficacy by modulating key pathways and inhibiting pro-inflammatory mediators. Volatile oils from *Myristica fragrans* and quercetin from *Amomum tsao-ko* exhibit potent radical-scavenging activity, contributing to their anti-inflammatory properties [46,83]. In particular, *Amomum tsao-ko* essential oil suppresses NO production and downregulates pro-inflammatory cytokines [84]. Luteolin derivatives from *Terminalia chebula* show strong antioxidant and DNA-protective effects [145], while gallic acid-enriched extracts of *Terminalia bellirica* activate the Akt/AMPK/Nrf2 pathway, leading to enhanced antioxidant enzyme expression and attenuation of LPS-induced inflammation [146].

Polysaccharides from *Choerospondias axillaris* demonstrate antioxidant and xanthine oxidase inhibitory activity, suggesting potential application in oxidative and gout-related inflammation [147]. In an atopic dermatitis model, *Terminalia chebula* extract downregulates cytokine expression and inhibits activation of STAT1/3 and NF-κB pathways, thereby exerting both anti-inflammatory and immunomodulatory effects [148].

Notably, hydroxysafflor yellow A—a major flavonoid from *Carthamus tinctorius*—exerts dual chondroprotective and anti-inflammatory actions in osteoarthritis. It prevents IκBα degradation and NF-κB p65 translocation, reducing IL-1β, COX-2, and MMP-13 expression. Concurrently, it activates the AMPK/SIRT1 axis, supporting mitochondrial energy homeostasis and antioxidant defense [149].

**Figure 8:**
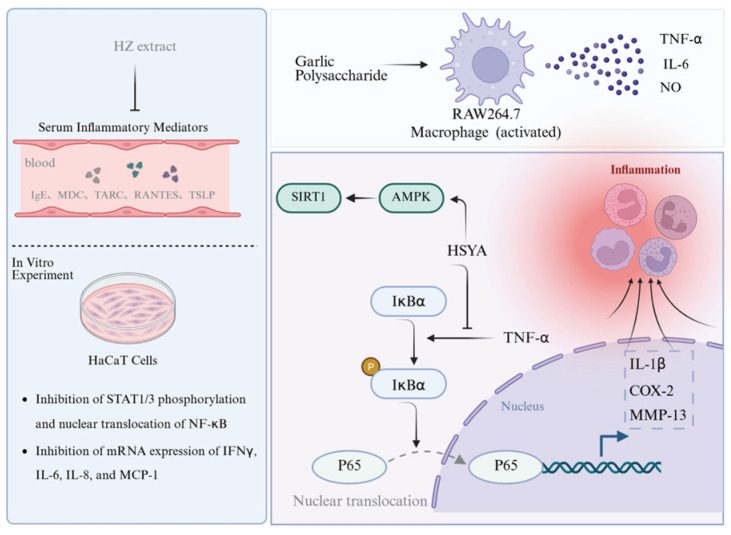
Immunomodulatory and Anti-Inflammatory Mechanisms of Active Compounds in Ershiwei Roudoukou Wan. Solid arrows indicate activation/promotion, whereas T-bar arrows indicate inhibition/blockade.

These diverse pharmacological profiles—ranging from macrophage activation and cytokine regulation to mitochondrial support and oxidative stress attenuation—reflect a converging theme: the immunological balance maintained by RDK20 constituents is achieved through coordinated, multi-level modulation rather than single-target intervention. Such a systems-level immunoregulatory strategy, as illustrated in Figure 8, may account for its broad applicability across inflammation-related conditions.

### 5.3. Antitumor Activities

Malignant tumors remain a leading cause of mortality worldwide, prompting sustained interest in natural product-based anticancer strategies, including traditional Chinese medicine and its bioactive constituents. Accumulating evidence indicates that multiple components of Ershiwei Roudoukou Wan exert antitumor effects through mechanistically distinct yet biologically complementary pathways.

β-Boswellic acid, a triterpenoid from Boswellia, exhibits pronounced antitumor activity through dual regulatory mechanisms. On the one hand, it activates the PERK/eIF2α/ATF4/CHOP signaling cascade, triggering endoplasmic reticulum (ER) stress and CHOP-dependent, caspase-mediated apoptosis. On the other, it suppresses the Wnt/β-catenin pathway by reducing Akt and GSK3β phosphorylation, thereby limiting nuclear β-catenin accumulation and downregulating oncogenic drivers such as c-Myc and Cyclin D1. Through this coordinated regulation of stress signaling and proliferative pathways, β-boswellic acid effectively inhibits osteosarcoma cell proliferation, invasion, and migration [150].

Essential oil from *Myristica fragrans* displays broad cytotoxic potential across multiple tumor types. It induces apoptosis in breast and colon cancer cells [32], suppresses cyclophosphamide-induced mutagenesis [151], and reduces the viability of hepatocellular carcinoma cells [81]. Consistent with these in vitro observations, crude extracts of nutmeg pericarp have shown antitumor efficacy in an Ehrlich ascites carcinoma mouse model [152], supporting a multi-level anticancer effect spanning both cellular and whole-organism contexts.

Sesquiterpene lactone–coumarins from *Ferula* species further expand the antineoplastic spectrum. Among them, (−)-Ferulasinkian A exhibits marked cytotoxicity against human pancreatic cancer cell lines, with IC_50_ values ranging from 4.57 ± 0.94 to 14.01 ± 1.03 μM. In CFPAC-1 cells, treatment induces classical apoptotic hallmarks, including chromatin condensation, DNA fragmentation, and cellular shrinkage, indicating direct engagement of programmed cell death pathways [133].

Beyond direct cytotoxicity, several constituents modulate antitumor immunity. Tannins from Phyllanthus emblica induce immunogenic cell death via PERK/ATF4/CHOP-dependent ER stress, leading to protein aggregation and the release of damage-associated molecular patterns such as calreticulin, ATP, and HMGB1. These signals activate type I interferon responses and CXCL9/10-mediated chemotaxis, thereby promoting CD8^+^ T-cell infiltration and reinforcing antitumor immune surveillance [153].

**Figure 9:**
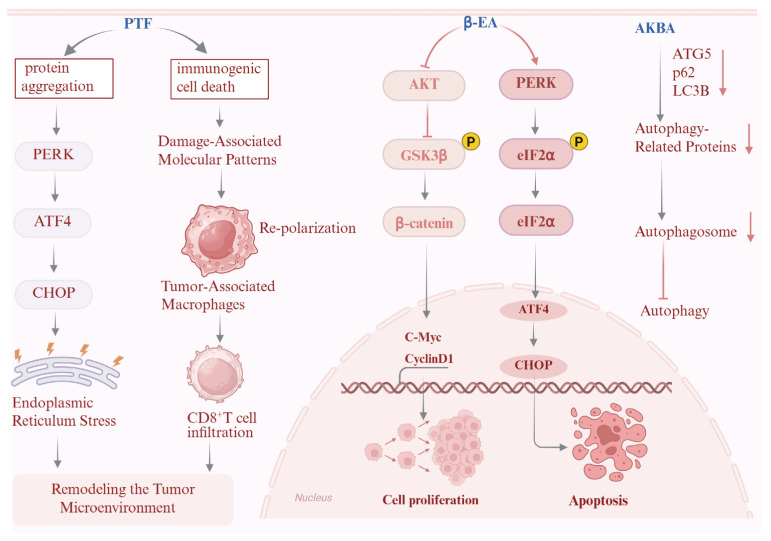
Anticancer mechanism of the active ingredients in Ershiwei Roudoukou Wan. Solid arrows indicate activation/promotion, whereas T-bar arrows indicate inhibition/blockade.

Closely related mechanisms are observed with other Boswellia-derived compounds. 3-O-acetyl-11-keto-β-boswellic acid (AKBA) suppresses autophagy in glioblastoma cells by downregulating autophagy-related proteins and disrupting ERK/mTOR and p53/mTOR signaling. By reducing autophagic flux, AKBA shifts the balance toward tumor cell death, highlighting autophagy inhibition as an additional anticancer strategy within this formula [154] (Figure 9).

Additional evidence supports a broader contribution from *Terminalia* and *Amomum* species. Tannins isolated from *Terminalia bellirica* demonstrate antitumor activity against hepatocellular carcinoma both in vitro and in vivo, largely through remodeling of the tumor immune microenvironment and enhancement of host antitumor responses [155]. Aqueous extracts of *Terminalia chebula* reduce skin tumor incidence in preclinical models [100], while *Amomum tsao-ko* extracts exhibit combined cytotoxic and antioxidant effects across multiple cancer cell lines [50]. Methanol extracts of Boswellia likewise show selective cytotoxicity toward leukemia cells [156].

Rather than converging on a single molecular target, these findings indicate that RDK20 constituents engage cancer through multiple entry points, including ER stress-mediated apoptosis, autophagy regulation, immune activation, redox modulation, and suppression of oncogenic signaling. This mechanistic diversity provides a plausible pharmacological basis for their complementary antitumor actions and underscores the value of RDK20 as a reservoir of structurally and functionally diverse anticancer leads.

### 5.4. Neuroprotective Effects

Cholinergic dysfunction and acetylcholine (ACh) deficiency are hallmark features of Alzheimer’s disease (AD)-related cognitive decline. Among the constituents of Ershiwei Roudoukou Wan, several compounds have shown neuroprotective effects through distinct mechanisms involving cholinergic enhancement, anti-inflammatory modulation, oxidative stress regulation, and mitochondrial protection.

Safflower yellow (SY), a major component of Carthamus tinctorius, exerts dual neuroprotective effects by enhancing cholinergic neurotransmission and activating neuronal survival pathways. It inhibits acetylcholinesterase (AChE) while upregulating choline acetyltransferase (ChAT), thereby increasing ACh levels in brain tissue. In parallel, SY promotes synaptic plasticity via activation of the CREB/BDNF/TrkB signaling axis, contributing to neuronal resilience [157].

Other ingredients exert protective effects by targeting neuroinflammation and oxidative injury. Kellerin, a bioactive compound from Ferula, attenuates cerebral infarction and edema in ischemic models by suppressing microglial activation and reducing neuroinflammatory responses [158]. Similarly, *Terminalia chebula* extract protects against methamphetamine-induced neurotoxicity and cognitive impairment through its antioxidant properties and ability to enhance neuronal viability [159]. Four ellagitannins isolated from T. chebula further act as AChE inhibitors, supporting their potential for cholinergic restoration in AD [99].

Additional neuroprotection is mediated through redox regulation. Acacia catechu extract improves neuronal survival under oxidative stress by reducing intracellular ROS and modulating calcium homeostasis-related signaling [160]. In vascular dementia models with hyperlipidemia, Calculus Bovis enhances cognitive performance by promoting antioxidant enzyme activity and balancing pro- and anti-apoptotic protein expression (Bax/Bcl-2) [161].

Neuroprotective actions have also been reported for several aromatic and phenolic compounds. Extracts of *Myristica fragrans*, particularly its lignan-rich ethyl acetate fraction, protect against neurodegenerative damage [86,162]. Essential oils from Syzygium aromaticum and *Ferula* species confer benefits via combined anti-inflammatory and antioxidant mechanisms [163]. Ethanol extract of Santalum album alleviates poly I:C-induced neuroinflammation by enhancing type I interferon responses and reducing expression of pro-inflammatory cytokines, including IL-6, CXCL8, and CCL2 [164].

Hydroxysafflor yellow A (HSYA), another key flavonoid from Carthamus tinctorius, exhibits neuroprotective effects beyond AD. In models of traumatic brain injury, it activates AMPK/mTOR signaling and promotes autophagy, facilitating neuronal recovery [165]. In cerebral ischemia–reperfusion injury, HSYA helps preserve mitochondrial function by maintaining membrane potential, inhibiting cytochrome c release, and preventing mitochondrial permeability transition pore opening [166].

Eugenol—a phenylpropanoid present in both *Myristica fragrans* and *Syzygium aromaticum*—has demonstrated therapeutic promise in transgenic AD mouse models. It enhances β-amyloid clearance by promoting microglial M2 polarization, reduces neuroinflammation, and limits Aβ plaque deposition, collectively improving cognitive performance [167]

**Figure 10:**
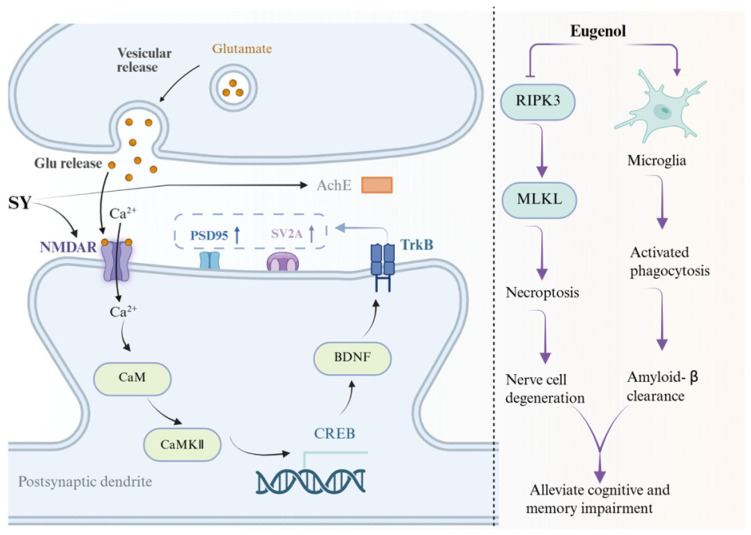
Neuroprotective mechanism of the active ingredients in Ershiwei Roudoukou Wan. Solid arrows indicate activation/promotion, whereas T-bar arrows indicate inhibition/blockade.

The diverse neuroprotective mechanisms—spanning cholinergic enhancement, inflammation resolution, mitochondrial stabilization, and oxidative defense—highlight the capacity of RDK20 constituents to intervene at multiple pathological nodes relevant to neurodegenerative progression (Figure 10).

### 5.5. Hepatoprotective Effects

Multiple constituents of Ershiwei Roudoukou Wan have shown protective effects against hepatic injury through mechanisms involving oxidative stress regulation, inflammation suppression, fibrosis inhibition, and apoptosis modulation.

Chebulinic acid, a key tannin from *Terminalia chebula*, exhibits hepatoprotective effects in models of chemical-induced liver damage, including t-BHP, acetaminophen (APAP), and carbon tetrachloride (CCl_4_). In hepatocyte cultures, it mitigates t-BHP-induced injury by suppressing ROS generation, lowering lactate dehydrogenase (LDH) release, and upregulating cytoprotective enzymes such as HO-1 and NQO1 via MAPK/Nrf2 pathway activation. Animal studies corroborate these findings, revealing significant improvements in serum ALT, AST, and MDA levels, along with enhanced SOD activity and improved liver histology, largely through Nrf2/HO-1 signaling engagement [168].

Artificial Calculus Bovis exerts hepatoprotective actions by targeting inflammatory and oxidative pathways. It blocks NF-κB signaling to reduce hepatic inflammation, while activating the Nrf2–GCLM/GCLC axis to promote antioxidant defenses. Additionally, it modulates the Bax/Bcl-2 ratio, thereby protecting hepatocytes from apoptosis [169].

**Figure 11:**
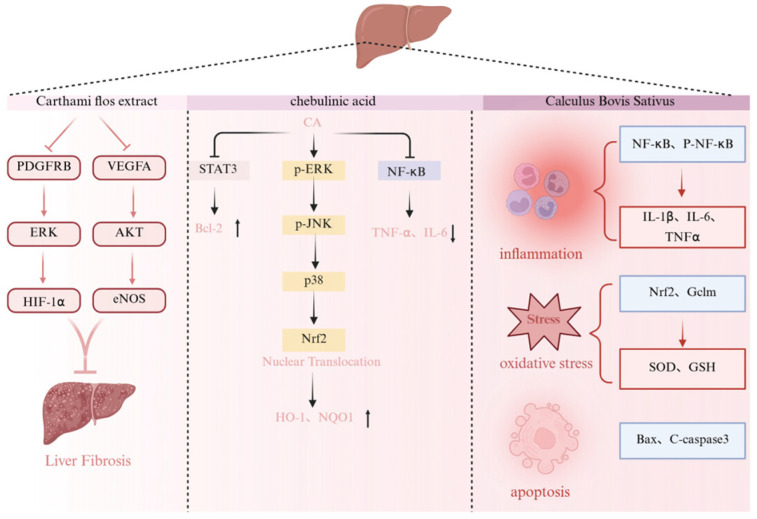
Hepatoprotective mechanism of the active ingredients in Ershiwei Roudoukou Wan. ↑ and ↓ indicate increase and decrease, respectively; solid arrows indicate activation/promotion; T-bar arrows indicate inhibition/blockade.

Extracts of *Carthami flos* further contribute to hepatic protection by counteracting fibrosis-related pathways. These extracts downregulate fibrosis-associated markers, inhibit collagen accumulation, and suppress aberrant angiogenesis. Mechanistically, these effects are mediated via inhibition of the PDGFRβ/ERK/HIF-1α and VEGFA/AKT/eNOS signaling cascades [170] (Figure 11).

Hydroxysafflor yellow A (HSYA), another major compound from *Carthamus tinctorius*, acts through dual mechanisms. It alleviates liver inflammation by inhibiting PI3K/Akt and STAT3/NF-κB pathways and simultaneously enhances antioxidant capacity by activating the Nrf2/Keap1 signaling axis [171]. The convergence of anti-inflammatory and antioxidant effects highlights its multifaceted hepatoprotective profile.

By targeting inflammatory cascades, redox homeostasis, fibrosis signaling, and apoptotic regulators, these agents demonstrate potential to restore hepatic integrity under diverse forms of injury. Such multi-level coordination suggests utility in both acute and chronic liver disease models (Figure 11).

### 5.6. Hypoglycemic Effects

Several bioactive constituents of Ershiwei Roudoukou Wan have demonstrated therapeutic potential in managing glucose and lipid metabolism disorders, primarily through digestive enzyme inhibition, enhancement of glucose uptake, and modulation of insulin sensitivity and inflammation.

Phenolic compounds from *Amomum tsao-ko* [93] and chebulagic acid from *Terminalia chebula* [23] exhibit significant α-glucosidase inhibitory activity, leading to improved postprandial glucose control in diabetic mouse models. Notably, phenolic constituents from *Myristica fragrans* not only inhibit α-amylase with greater potency than acarbose [172], but also contain phenylpropanoids capable of enhancing glucose uptake in muscle cells [173], suggesting a dual role in glycemic regulation.

Polar methanol fractions of A. tsao-ko have been reported to exert both hypoglycemic and hypolipidemic effects by targeting multiple digestive enzymes [27]. Similarly, extracts of *Acacia catechu* exhibit dual inhibition of α-glucosidase and α-amylase, reinforcing their value in carbohydrate digestion control [90]. *Allium sativum* (garlic) extract improves glucose metabolism in diabetic rats, likely through suppression of systemic pro-inflammatory cytokines and enhanced insulin responsiveness [174].

Beyond enzymatic and inflammatory pathways, several compounds contribute to broader metabolic regulation. Gallic acid, a prominent phenolic from *Phyllanthus emblica*, promotes adipocyte differentiation and lipid droplet formation in vitro, while upregulating PPAR-γ and GLUT4 expression. In vivo, gallic acid activates the Akt signaling pathway, reduces hyperglycemia in both db/db and fructose-induced insulin resistance models, and improves multiple metabolic indicators, including body weight, hepatic steatosis, and plasma TNF-α levels [175]. These findings suggest a capacity to address insulin resistance at both molecular and systemic levels.

Instead of focusing solely on glucose-lowering effects, these compounds act across digestive, inflammatory, and cellular signaling pathways that collectively reshape the metabolic landscape. Their capacity to address upstream dysregulation makes them particularly relevant to early-stage intervention in metabolic syndrome and its complications.

### 5.7. Gastrointestinal Protective Effects

Several constituents of Ershiwei Roudoukou Wan have demonstrated protective effects on the gastrointestinal system, with actions spanning anti-inflammatory modulation, oxidative stress attenuation, microbiota regulation, and intestinal barrier reinforcement.

Myristicin, a phenylpropanoid from *Myristica fragrans*, alleviates ulcerative colitis by reducing ER stress-induced apoptosis. It downregulates GRP78 and CHOP expression while activating the Nrf2/HO-1 axis to enhance antioxidant defenses [176]. In addition, aqueous extracts of M. fragrans suppress pro-inflammatory cytokine production and reduce disease severity in DSS-induced colitis models [177].

**Figure 12:**
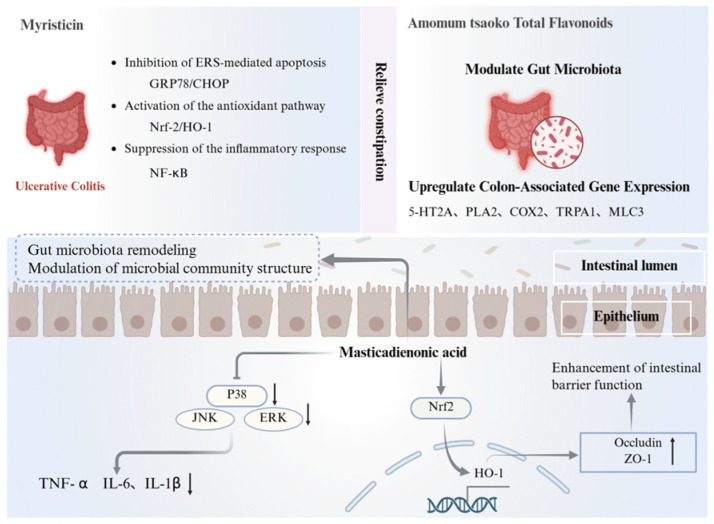
Gastrointestinal mechanism of the active ingredients in Ershiwei Roudoukou Wan. ↑ and ↓ indicate increase and decrease, respectively; solid arrows indicate activation/promotion; T-bar arrows indicate inhibition/blockade.

Masticadienonic acid (MDA) from Boswellia exerts multifaceted protective effects. It inhibits MAPK (ERK, JNK, p38) and NF-κB signaling, thereby reducing inflammatory mediator release. Concurrently, MDA activates the Nrf2 pathway and enhances intestinal barrier integrity by upregulating tight junction proteins ZO-1 and occludin, reducing epithelial permeability and mucosal damage [64,178] (Figure 12).

Phyllanthus emblica-derived polysaccharides (PEPs) also show anti-colitic activity, particularly in TNBS-induced models. PEPs suppress oxidative and inflammatory markers, increase anti-inflammatory cytokine levels, and restore gut microbial balance while modulating inflammation-related signaling cascades [125]. Regulation of gut motility and microbiota composition has also been observed. Flavonoids from *Amomum tsao-ko* relieve constipation by enhancing intestinal transit and altering microbial composition—characterized by increased beneficial bacteria, elevated serum 5-HT, and modulation of prostaglandin metabolites and colonic gene expression [94]. Likewise, *Aquilaria sinensis* methanol extract exhibits antispasmodic effects on gastrointestinal smooth muscle, potentially relieving abdominal cramping and spasm-associated disorders [64].

Their ability to act at the level of both symptom relief and mucosal repair may be particularly valuable for managing relapsing–remitting gastrointestinal disorders such as colitis or functional bowel diseases.

### 5.8. Other Pharmacological Activities

Beyond its primary cardiovascular, neuroprotective, and immunomodulatory properties, Ershiwei Roudoukou Wan also contains constituents with promising activities in metabolic regulation, analgesia, antimicrobial defense, and neuroinflammation resolution.

Metabolic Regulation and Anti-Obesity Effects

Phyllanthus emblica and its major component, gallic acid, reduce adiposity by inhibiting key adipogenic regulators (PPARγ, C/EBPα, FABP4) and inducing adipocyte apoptosis via BAX upregulation, BCL2 suppression, and caspase-3 activation [179]. These effects suggest potential in the management of obesity and metabolic syndrome.

Analgesic Activity and Autophagy Modulation

M. fragrans methanol extract exhibits both central and peripheral analgesic effects, attributed to non-selective opioid receptor engagement and modulation of the L-arginine/nitric oxide pathway [180]. Additionally, its constituent myrifratin D has been shown to influence autophagy by increasing LC3-II and p62 expression in vitro, suggesting regulatory effects on cellular stress responses [181].

Antimicrobial and Antiviral Activity

Ethanol and ethyl acetate fractions of *Amomum tsao-ko* show potent antibacterial activity against Klebsiella pneumoniae [182]. Likewise, bilirubin from Calculus Bovis exhibits concentration-dependent inhibition of Staphylococcus aureus, Pseudomonas aeruginosa, S. epidermidis, and Escherichia coli [112]. *Terminalia bellirica* extracts demonstrate strong antimicrobial activity against multidrug-resistant pathogens, including MRSA, Acinetobacter baumannii, and Pseudomonas aeruginosa, with MICs as low as 0.25 mg/mL [183].

In parallel, Boswellia resin and its principal compound β-boswellic acid exhibit antiviral activity against HSV-1 by downregulating viral gene expression at multiple stages (ICP27, DNA polymerase, gD). They also modulate the host immune response by shifting the Th1/Th2 cytokine profile, downregulating IFN-γ and IL-12 while upregulating IL-4 and IL-10. Inhibition of iNOS expression and NO production further contributes to reduced inflammatory tissue damage [184].

Neuropathic Pain and Neuroinflammation

Ferulic acid alleviates sciatic nerve injury-induced allodynia by downregulating pro-inflammatory cytokines and pain-associated ion channels (TRPA1, TRPV1). It promotes microglial polarization toward the anti-inflammatory M2 phenotype via RhoA/p38 MAPK signaling, facilitating inflammation resolution in chronic pain states [185]. While these findings span a broad range of biological systems, they converge on a common theme: multi-target modulation of complex disease networks. These effects not only suggest therapeutic value in diverse conditions but also warrant further investigation into combinatory use, disease-specific targeting, and the broader clinical applicability of RDK20 beyond its traditional indications.

**Table 1:** Pharmacological Activities of Bioactive Components in Ershiwei Roudoukou Wan.

Source Plants	Extracts/ Compounds	Pharmacological Effects	Model/Cell Line	Results	Doses	Ref.
*Myristica fragrans Houtt*	Volatile Oil	Anticancer	Novikoff rat hepatoma cells	Cx43 ↓	79.4 μg/mL	[81]
	Myristicin	Gastrointestinal protection	Ulcerative colitis rat model	GRP78 ↓, CHOP ↓, Nrf-2 ↑, HO-1 ↑, MPO ↓,	150 mg/kg	[176]
	Methanol extract	Anticancer	Ehrlich ascites carcinoma model	hemoglobin ↑, hematocrit ↑	50 and 100 mg/kg	[152]
	Ethanol extract	Hypoglycemic	Rat skeletal muscle L6 cells	p-AMPK Glucose Uptake ↑	10 μg/mL	[173]
	Myrifratin D	Autophagy regulation	Human embryonic kidney 293 cells	LC3-II ↑, p62 ↑	10 and 20 μM	[181]
*Dalbergia odorifera*	Flavonoids	Cardiovascular protection	Myocardial ischemia model	CK ↓, ALT ↓, GSH ↑, Na+-K+-ATPases ↑	100 mg/kg	[136]
*Choerospondias axillaris*	Methanol extract	Lipid-lowering	Hyperlipidemia rat model	LDL-C ↓, VLDL-C ↓, TC ↓, TG ↓,	200 mg/kg, 400 mg/kg	[186]
*Terminalia chebula Retz*	Methanol extract	Cardiac protection	Cardiotoxicity model induced by doxorubicin and isoproterenol	CK-MB ↓, MDA ↓, GSH ↑,	250 mg/kg	[139]
	50% ethanol extract	Neuroprotection	Cognitive impairment model induced by methamphetamine	ERK ↑, Nrf2 ↑, MDA ↓, γ-H2AX ↓	10 mg/kg, 100 mg/kg	[159]
	Aqueous extract	Anti-inflammatory	Mouse atopic dermatitis model	IgE ↓, MDC ↓, TARC ↓, RANTES ↓, TSLP ↓, STAT1/3 ↓, NF-κB ↓	100 mg/kg	[148]
*Syzygium aromaticum*	Clove saponin	Cardiac protection	Type 2 diabetes rat model	IL-1β ↓, TNF-α ↓, BNP ↓, CK-MB ↓, cTnI ↓	50 mg/kg	[140]
	Eugenol	Neuroprotection	5xFAD transgenic mouse model of Alzheimer’s disease	Aβ ↓, IL-6 ↓, IL-1β ↓, MACRO ↑, CD36 ↑	10 mg/kg, 30 mg/kg	[167]
	Phenolic compounds	Hypoglycemic	Diabetic mouse model	α-glucosidase activity ↓	100 mg/kg, 200 mg/kg	[93]
	Total flavonoids	Gastrointestinal regulation	Slow transit constipation model	5-HT ↑, 5-HT2A ↑, PLA2 ↑, COX2 ↑, TRPA1 ↑, MLC3 ↑, iNOS ↓	150 mg/kg	[94]
*Terminalia belerica Roxb*	Polyphenols	Anti-inflammatory	LPS-induced inflammatory mouse model	NQO1 ↑, GCLM ↑, TNF-α ↓, IL-6 ↓	400 mg/kg	[146]
	Tannins	Anticancer	Hepa1-6 orthotopic hepatocellular carcinoma mouse model	Fn1 ↓, Col1a1 ↓, Acta2 ↓, IL-6 ↑, TNF-α ↑, IL-1β ↑, iNOS ↑	2 g/kg	[155]
*Carthamus tinctorius*	Hydroxysafflor yellow A	Anti-inflammatory	Rat chondrocytes	IL-1β ↓, PTGS2 ↓, MMP-13 ↓, COL2A1 ↑, ACAN ↑	10 µM	[149]
	Hydroxysafflor yellow A	Neuroprotection	Mouse model of impairment in learning, memory acquisition, consolidation, and retrieval	ChAT ↑, Ach ↑, AChE ↓, BDNF ↑, PSD95 ↑, SV2A ↑, NMDAR2B ↑	30 and 100 mg/kg	[157]
	Hydroxysafflor yellow A	—	Traumatic brain injury model	IL-18 ↓, IFN-γ ↓, GFAP ↓, NLRP3 ↓, ASC ↓, LC3 II/LC3 I ↑, P62 ↓	9 mg/kg	[165]
	Aqueous extract	Hepatoprotective	CCl_4_-induced liver fibrosis model	ALT ↓, AST ↓, ALP ↓, γ-GT ↓, α-SMA ↓, HYP ↓, Col-IV ↓, PDGFRB ↓, p-MEK ↓, p-ERK1/2 ↓, HIF-1α ↓, VEGFA ↓, CD31 ↓, CD34 ↓, vWF ↓	1, 2, 4 g/kg	[170]
	Hydroxysafflor yellow A	Hepatoprotective	Ethanol-induced liver injury model	Nrf2 ↑, HO-1 ↑, NQO-1 ↑, GCLM ↑, Keap1 ↓, p-PI3K ↓, p-Akt ↓, p-mTOR ↓, PPARα ↑	2.5 mg/kg, 7.5 mg/kg	[171]
*Boswellia carterii*	β-Oleanolic acid	Anticancer	Human osteosarcoma cells	Bcl-2 ↓, Bax ↑, Cyclin D1 ↓, c-Myc ↓, β-catenin ↓, Bip/GRP78 ↑, p-PERK ↑, ATF4 ↑, CHOP ↑	7.5, 15, and 30 μM	[150]
	3-O-acetyl-11-keto-β-boswellic acid	—	U87-MG orthotopic glioma model	ATG5 ↓, P62 ↓, LC3-II ↓, ATG3 ↓, ATG7 ↓, ATG12 ↓, ATG16 ↓	100 mg/kg	[154]
	masticadienonic acid	Gastrointestinal regulation	Acute colitis mouse model	TNFα ↓, IL-1β ↓, IL-6 ↓, ZO-1 ↑, occludin ↑	10 mg/kg, 100 mg/kg	[178]
	β-Oleanolic acid	Antiviral	HSV-1	ICP27 ↓, DNA-Pol ↓, TNF-α ↓, IL-1β ↓, IL-6 ↓	30 μg/mL	[184]
*Ferula Sinkiangensis*	Ferulasinkian A	Anticancer	Human pancreatic cancer cell line	Promotes nuclear condensation and fragmentation	10 μM	[133]
	Kellerin	Neuroprotection	Middle cerebral artery occlusion (MCAO) model	TNF-α ↓, IL-6 ↓, iNOS ↓, p-IκBα ↓, IκBα ↑	3.5, 7, 14 mg/kg	[158]
	Ferulic acid	Analgesic	Ethanol-induced alcoholic liver injury model	PGE2 ↓, SP ↓, CRP ↓, IBA-1 ↓, RhoA ↓, iNOS ↓, IL1β ↓, COX2 ↓, Rock1 ↓, TRPV1 ↓, TRPA1 ↓, p-p38MAPK ↓	50 mg/kg/bid	[185]
*Phyllanthus emblica*	60% ethanol extract	—	Lewis lung cancer cells, A549 cells, and RAW 264.7 cells	Tgf-β1 ↓, Cd 206 ↓, IL-6 ↓, Ccl5 ↑, Cxcl 9 ↑, PERK/ATF4/CHOP ↑	320 μg/mL	[153]
	ethanol extract	Anti-obesity	3T3-L1 preadipocytes	PPARγ ↓, CEBPα ↓, FABP4 ↓, BAX ↑, BCL2 ↓	10, 25, 50, 100, and 200 µg/mL	[179]
	Gallic acid	Hypoglycemic	—	PPAR-γ ↑, Glut4 ↑	2–20 µM	[175]
*Senegalia catechu*	Aqueous extract	Neuroprotection	H_2_O_2_-induced damage in human SH-SY5Y neuroblastoma cells	ROS ↓, MDA ↓, Bax/Bcl-2 ratio ↓, caspase-3 ↓	100 and 200 µg/mL	[160]
	ethanol extract	Hypoglycemic	3T3-L1 adipocyte model	α-glucosidase (IC50: 0.3353 ± 0.1215 μg/mL)		[90]
*Calculus Bovis*	In vitro cultured bezoar	Neuroprotection	Hyperlipidemia-related vascular dementia model	Bax ↓, Bcl-2 ↑, SOD ↑, NO ↑, MDA ↓	0.06, 0.12 g/kg	[161]
		Hepatoprotective	Estrogen-induced cholestasis model	TNFα ↓, IL-1 ↓, IL-6 ↓, MDA ↓, GSH ↑	150 mg/kg	[169]
*Santalum album*	ethanol extract	Neuroprotection	Human neuroblastoma cell line	TLR2 ↑, TLR4 ↑, IFN-β ↑, IFN-α ↑, IL-6 ↓, CXCL8 ↓, CCL2 ↓, IP-10 ↓	50 μg/mL, 200 μg/mL	[164]

Note: ↑ and ↓ indicate increase and decrease.

### 5.9. Summary

In summary, existing pharmacological studies have established a multisystem pharmacological profile for RDK20, supported by evidence across cardiovascular, neural, hepatic, immune, and metabolic domains. However, current research remains largely fragmented, with most studies isolating single components or focusing on individual pathways. To fully understand the therapeutic scope and optimize clinical translation, future investigations should emphasize the integrated effects of the complete formulation, particularly in terms of synergistic interactions and dynamic multi-target regulation.

## 6. Discussion

Ershiwei Roudoukou Wan is a classical multi-herbal prescription rooted in traditional Tibetan medicine [1,187], distinguished by its complex composition and broad therapeutic indications. This review integrates bibliometric analysis, clinical observations, and phytochemical evidence to provide a structured summary of current research progress. Collectively, the available data suggest that its pharmacological effects are more likely derived from the coordinated actions of multiple compound classes, rather than the dominance of a single bioactive constituent.

Chemically, existing studies indicate that flavonoids, organic acids, tannins, polysaccharides, and volatile constituents form the major material basis of Ershiwei Roudoukou Wan. These chemical classes are widely distributed across the component herbs and exhibit diverse physicochemical characteristics. Flavonoids and organic acids are frequently implicated in antioxidant and anti-inflammatory pathways [188]; tannins and polysaccharides are associated with immunomodulatory and antimicrobial effects [189,190]; and volatile components—particularly terpenoids and phenylpropanoids—may contribute to the aromatic properties and central nervous system modulation described in traditional usage [191]. Although such associations are supported by numerous in vitro and in vivo studies, they currently represent functional correlations rather than definitive mechanistic conclusions.

In interpreting the pharmacological relevance of this formula, it is important to recognize the heterogeneity and limitations of the current evidence base. Most published data are derived from studies on individual herbs or isolated compounds, utilizing cellular models or animal experiments. By contrast, systematic investigations of the full prescription remain scarce and are mostly limited to retrospective clinical observations lacking standardized outcome measures. This disconnect is emblematic of a broader challenge in the modernization of traditional multi-component therapies: mechanistic and chemical studies often progress more rapidly than formulation-level in vivo validation.

The bibliometric findings further underscore a research imbalance, with individual herbs such as Carthamus tinctorius, Allium sativum, Syzygium aromaticum, and Aquilaria sinensis receiving disproportionate attention compared to the complete formulation. While such reductionist approaches have yielded valuable insights into phytochemical diversity and pharmacological potential, they risk over-extrapolation. Given the potential for herb–herb interactions to alter both pharmacokinetics and bioactivity, the translation of single-herb findings to multi-herb systems should be undertaken cautiously and validated through integrated studies [192].

Clinically, Ershiwei Roudoukou Wan has been used in the management of conditions such as cholecardia syndrome, disorders associated with “Ninglong”, and various gynecological diseases [1]. However, most of the clinical reports are descriptive in nature, lacking control groups, quantitative assessments, or long-term follow-up. As such, while these studies offer preliminary support for efficacy and safety, they do not establish therapeutic specificity or mechanistic clarity.

In conclusion, the current literature suggests that Ershiwei Roudoukou Wan possesses a chemically and pharmacologically diverse foundation that aligns with its traditional indications. However, the evidence remains fragmented and predominantly preclinical. Future investigations should prioritize integrative approaches that combine advanced chemical profiling, systems pharmacology, well-characterized animal models, and rigorously designed clinical trials. Such efforts will be essential to clarify the therapeutic mechanisms, validate clinical efficacy, and support the rational modernization of this traditional Tibetan formula.

## Data Availability

No new data were created or analyzed in this study.

## Supplementary Materials

The following supporting information can be downloaded at: https://www.mdpi.com/article/10.3390/ph19010052/s1, Table S1: Chemical Constituents of Individual Herbs in Ershiwei Roudoukou Wan.

## Abbreviations

The following abbreviations are used in this manuscript:

PGC1α	Proliferator-activated receptor-gamma coactivator-1 alpha
SIRT3	sirtuins-3
TLR4	Toll-like receptor-4
MyD88	myeloid differentiation primary response-88
NF-κB	nuclear factor-kappa B
ATP	Adenosine Triphosphate
ROS	reactive oxygen species
BAX	Bcl-2-associated X protein
Bcl2	B-cell lymphoma 2
MnsOD	manganese superoxide dismutase
Mito ROS	mitochondrial reactive oxygen species
Mito ∆Ψ	mitochondrial membrane potential
GPX	glutathione peroxidase
CK-MB	creatine kinase-myocardial band
IL-6	interleukin-6
IL-1β	interleukin-1 beta
lgE	Immunoglobulin E
MDC	Macrophage-Derived Chemokine
TARC	Thymus and Activation-Regulated Chemokine
RANTES	Regulated on Activation, Normal T-cell Expressed and Secreted
TSLP	Thymic Stromal Lymphopoietin
STAT1/3	Signal Transducer and Activator of Transcription 1/3
IFN-γ	Interferon-γ
IL-8	interleukin-8
MCP-1	Monocyte Chemoattractant Protein-1
AMPK	Adenosine Monophosphate-Activated Protein Kinase
HYSA	Hydroxysafflor yellow A
MMP-13	matrix metalloproteinase-13
COX-2	cyclooxygenase-2
IκBα	Inhibitor of Kappa B Alpha
NO	nitric oxide
PERK	protein kinase R-like endoplasmic reticulum kinase
ATF4	activating transcription factor
CHOP	C/Enhancer-Binding Protein Homologous Protein
β-EA	β-boswellic acid
AKT	Protein Kinase B
GSK3β	Glycogen Synthase Kinase 3 Beta
C-Myc	Cellular Myelocytomatosis oncogene
eIF2α	Eukaryotic Translation Initiation Factor 2 Subunit Alpha
AKBA	3-O-acetyl-11-keto-b-boswellic acid
ATG5	Autophagy Related 5
LC3B	Microtubule-associated protein 1A/1B-light chain 3B
SY	Safflower yellow
NMDAR	N-methyl-D-aspartate receptor
CaM	Calmodulin
CaMK	Calmodulin-dependent kinase
CREB	Cyclic AMP-responsive element-binding protein
BDNF	Brain-derived neurotrophic factor
TrkB	Tropomyosin receptor kinase B
AChE	Acetylcholinesterase
PSD95	Postsynaptic density 95
SV2A	Synaptic vesicle glycoprotein 2A
RIPK3	Receptor-Interacting Protein Kinase 3
MLKL	Mixed Lineage Kinase Domain-Like protein
PDGFRB	platelet-derived growth factor receptor beta
VEGFA	vascular endothelial growth factor A
ERK	extracellular regulated protein kinases
HIF-1α	hypoxia inducible factor 1 alpha
eNOS	endothelial nitric oxide synthase
STAT3	Signal Transducer and Activator of Transcription 3
p-ERK	phospho-extracellular regulated protein kinases
p-JNK	phospho-c-Jun N-terminal Kinase
Nrf2	Nuclear factor erythroid 2-related factor 2
HO-1	Heme oxygenase-1
NQO1	NAD(P)H quinone oxidoreductase-1
Gclm	glutamate cysteine modified linker
SOD	Superoxide dismutase
GSH	glutathione
5-HT2A	5-hydroxytryptamine receptor 2A
PLA2	Phospholipase A2
TRPA1	Transient receptor potential A1
MLC3	Myosin light chain 3
JNK	c-Jun N-terminal Kinase
ZO-1	Zonula occludens-1
Cx43	Connexin 43
GRP78	Glucose-Regulated Protein 78
MPO	Myeloperoxidase
p-AMPK	phosphorylated 5′ AMP-activated protein kinase
LC3-II	Microtubule-associated protein 1A/1B-light chain 3-phosphatidylethanolamine conjugate
CK	Creatine kinase
ALT	Alanine aminotransferase
LDL-C	Low-density lipoprotein cholesterol
VLDL-C	Very-low-density lipoprotein cholesterol
TC	Total cholesterol
TG	Triglycerides
MDA	Malondialdehyde
γ-H2AX	Phosphorylated histone H2AX
TNF-α	Tumor necrosis factor-alpha
BNP	B-type natriuretic peptide
cTnI	Cardiac troponin I
Aβ	Amyloid-beta peptide
MACRO	Macrophage receptor with collagenous structure
CD36	Cluster of Differentiation 36
IgG	Immunoglobulin G
iNOS	Inducible nitric oxide synthase
p-p65	Phosphorylated NF-κB p65 subunit
5-HT	5-hydroxytryptamine
Fn1	Fibronectin 1
Col1a1	Collagen type I alpha 1 chain
Acta2	Actin, alpha-2 smooth muscle
PTGS2	Prostaglandin-endoperoxide synthase 2
COL2A1	Collagen type II alpha 1 chain
ACAN	Aggrecan
ChAT	Choline acetyltransferase
Ach	Acetylcholine
NMDAR2B	N-methyl-D-aspartate receptor subunit 2B
GFAP	Glial fibrillary acidic protein
NLRP3	NOD-like receptor family pyrin domain-containing 3
ASC	Apoptosis-associated speck-like protein containing a CARD
AST	Aspartate aminotransferase
ALP	Alkaline phosphatase
γ-GT	Gamma-glutamyl transferase
α-SMA	Alpha-smooth muscle actin
HYP	Hydroxyproline
Col-IV	Collagen type IV
p-MEK	Phosphorylated mitogen-activated protein kinase kinase
p-ERK1/2	Phosphorylated extracellular signal-regulated kinases 1/2
CD31	Cluster of differentiation 31
CD34	Cluster of differentiation 34
vWF	von Willebrand factor
Keap1	Kelch-like ECH-associated protein 1
NQO-1	NAD(P)H quinone oxidoreductase 1
p-PI3K	Phosphorylated phosphatidylinositol-3-kinase
p-Akt	Phosphorylated protein kinase B
p-mTOR	Phosphorylated mechanistic target of rapamycin
PPARα	Peroxisome proliferator-activated receptor alpha
ICP27	Infected-cell protein 27
DNA-Pol	DNA polymerase
p-IκBα	Phosphorylated inhibitor of nuclear factor kappa B alpha
PGE2	Prostaglandin E2
SP	Substance P
CRP	C-reactive protein
IBA-1	Ionized calcium-binding adapter molecule 1
RhoA	Ras homolog family member A
Rock1	Rho-associated coiled-coil-containing protein kinase 1
TRPV1	Transient receptor potential vanilloid 1
p38MAPK	p38 mitogen-activated protein kinases
Tgf-β1	Transforming Growth Factor-β1
Cd206	Mannose Receptor C-type 1
Ccl5	C-C motif chemokine ligand 5
Cxcl9	C-X-C motif chemokine ligand 9
PPARγ	Peroxisome proliferator-activated receptor gamma
CEBPα	CCAAT/enhancer-binding protein alpha
FABP4	Fatty acid-binding protein 4
PPAR-γ	Peroxisome proliferator-activated receptor gamma
Glut4	Glucose transporter type 4
IL-1	Interleukin-1
TLR2	Toll-like receptor 2
IFN-β	Interferon-beta
IFN-α	Interferon-alpha
CXCL8	C-X-C motif chemokine ligand 8
CCL2	C-C motif chemokine ligand 2
IP-10	Interferon-gamma-inducible protein 10
