# Supplementary material for: Analysis of Research Progress on the Chemical Constituents and Pharmacological Activities of Er-Shiwei Roudoukou Wan
Source: Pharmaceuticals (Basel). 2025 Dec 25;19(1):52. doi: 10.3390/ph19010052 (PMC12844774; doi:10.3390/ph19010052)
Supplement: Supplementary file 1 [file pharmaceuticals-19-00052-s001.zip › pharmaceuticals-4021226-supplementary.pdf]

Table .S1. Chemical Constituents of Individual Herbs in Ershiwei Roudoukou Wan

Compounds	Formula	Source	Ref
**Flavonoids**			
Daidzein	$C_{15}H_{12}O_5$	JX	[1]
Liquiritigenin	$C_{15}H_{12}O_5$	JX	[1, 2]
Eriodictyol	$C_{15}H_{10}O_6$	JX, HZ	[1, 3]
Kaempferol	$C_{15}H_{10}O_6$	DX, JX, ZHX, EC, MHZ, GZ	[1, 4-8]
koparin	$C_{27}H_{30}O_{14}$	JX	[1]
xenognosinB	$C_{16}H_{12}O_5$	JX	[1]
Pinocembrin	$C_{15}H_{12}O_4$	JX	[1]
Dalbergin	$C_{15}H_{12}O_5$	JX	[1, 2, 9]
Isoliquiritigenin	$C_{27}H_{30}O_{14}$	JX	[1, 2, 9, 10]
BiochaninA	$C_{16}H_{12}O_5$	JX	[1]
Aquilarisinin	$C_{25}H_{30}NaO_{14}$	CX	[11]
Aquilarisin	$C_{22}H_{21}O_{13}$	CX	[11]
4′-O-Geranyltricin	$C_{15}H_{10}O_6$	CX	[12]
Luteolin	$C_{21}H_{20}O_{11}$	JX, DX, MHZ	[2, 13, 14]
Kaempferide	$C_{16}H_{20}O_6$	DX	[13]
Syringin	$C_{15}H_{14}O_6$	DX	[13]
Catechin	$C_{15}H_{14}O_6$	GZ, EC, DX, CG	[5, 13, 15, 16]
isocartormin	$C_{27}H_{29}NO_{13}$	HH	[17]

Compounds	Formula	Source	Ref
(S)-flavogallonic acid	$C_{27}H_{22}O_{18}$	HZ	[18]
3′-hydroxyhydroxysafflor yellowA	$C_{40}H_{30}O_{15}$	HH	[19]
HydroxysaffloryellowB	$C_{27}H_{32}O_{16}$	HH	[20]
HydroxysaffloryellowC	$C_{27}H_{32}O_{16}$	HH	[20]
Scutellarein	$C_{15}H_{10}O_{6}$	MHZ, HZ	[3, 7]
Genistein	$C_{19}H_{18}O_{11}$	MHZ	[7]
Vaccarin	$C_{32}H_{38}O_{19}$	JX	[21]
Mangiferin	$C_{16}H_{12}O_{6}$	MHZ	[7]
Homoplantaginin	$C_{22}H_{22}O_{11}$	JX	[21]
Isoorientin	$C_{15}H_{10}O_{5}$	MHZ	[7]
Apigenin	$C_{15}H_{14}O_{6}$	MHZ	[7]
Epicatechin	$C_{21}H_{20}O_{12}$	CG, EC, MHZ	[5, 7, 15, 22-24]
Hyperin	$C_{15}H_{10}O_{6}$	GZ, CG	[15, 16]
Butrin	$C_{15}H_{12}O_{5}$	JX,	[1, 10]
Isoquercetin	$C_{21}H_{20}O_{11}$	HH, CG	[25]
Afzelin	$C_{21}H_{20}O_{11}$	EC	[5, 26]
Epiafzelin	$C_{15}H_{14}O_{5}$	EC	[5]
3,3',4',7-Tetrahydroxyflavan	$C_{15}H_{14}O_{5}$	EC	[27]
HydroxysaffloryellowA	$C_{27}H_{32}O_{16}$,	HH	[26]
Latifolin	$C_{21}H_{20}O_{12}$	JX	[2, 10]
Isoquercitrin	$C_{15}H_{10}O_{7}$	CG	[28]
Quercitrin	$C_{15}H_{10}O_{8}$	CG	[28]

Compounds	Formula	Source	Ref
Myricetin	$C_{15}H_{18}O_6$	CG	[28]
Kaempferol	$C_{40}H_{64}O$	CG	[28]
Daucosterol	$C_{35}H_{60}O_6$	RDK, CG	[22, 29]
2-(2-Phenylethyl) chromone	$C_{17}H_{14}O_2$	CX	[30]
Astragaloside	$C_{15}H_{24}O$	JX	[10]
Baimuxualene	$C_{17}H_{16}O_5$	JX	[31]
Ononin	$C_{15}H_{12}O_5$	JX	[32]
Naringenin	$C_{15}H_{10}O_6$	JX, YGZ	[1, 2, 33]
Butin	$C_{15}H_{12}O_5$	JX	[1, 9]
vestitone	$C_{15}H_{10}O_5$	JX	[1]
Genistein	$C_{15}H_{10}O_5$	JX	[1]
Irisflorentin	$C_{21}H_{20}O_{10}$	JX	[1]
Formononetin	$C_{16}H_{12}O_4$	JX	[1, 10, 31]
3'-O-Methylviolanone	$C_{15}H_{10}O_6$	JX	[1]
Prunetin	$C_{16}H_{12}O_5$	JX	[1]
Medicarpin	$C_{15}H_{12}O_5$	JX	[1, 10]
3′-O-Geranylpolloin	$C_{15}H_{10}O_8$	CX	[12]
Myricetin	$C_{21}H_{20}O_{12}$	ZHX, DX, YGZ, GZ	[4, 8, 13, 33]
Isoquercitrin	$C_{16}H_{20}O_6$	ZHX, DX	[4, 13]
Syringaresinol-4'-O-β-D-glucopyranoside	$C_{15}H_{12}O_6$	DX	[13]
Taxifolin	$C_{27}H_{30}O_{16}$	CG	[15]
Rutin	$C_{21}H_{20}O_{11}$	DK, CG	[15, 28, 34]
Quercitrin	$C_{21}H_{20}O_{11}$	EC, CG	[5, 15]

Compounds	Formula	Source	Ref
Pinocembrin	$C_{15}H_{12}O_4$	MHZ	[14]
Parthenin	$C_{16}H_{12}O_6$	MHZ	[14]
(-)-Epibenin	$C_{15}H_{12}O_6$	CG	[35]
Taxifolin	$C_{27}H_{30}O_{14}$	CG	[28]
Naringin	$C_{15}H_{10}O_7$	CG	[28]
Quercetin	$C_{15}H_{10}O_7$	DX, CG, GZ, HZ, AW, YGZ	[6, 16, 22, 28, 36-38]
Vitexin	$C_{21}H_{20}O_{10}$	HZ	[3]
Naringin	$C_{27}H_{32}O_{14}$	HZ	[3]
Morin	$C_{15}H_{10}O_7$	CG	[28]
Isorhamnetin	$C_{16}H_{12}O_7$	CG	[28]
5,7-Diacetylchrysin	$C_{19}H_{14}O_6$	RDK	[39]
6-Hydroxy-2-phenylethylchromone	$C_{17}H_{14}O_3$	CX	[30]
Quercetin-3,4'-di-O-glucoside	$C_{27}H_{30}O_{17}$	MHZ	[7]
Melanettin	$C_{16}H_{12}O_5$	JX	[10]
Alpinin	$C_{16}H_{14}O_4$	JX	[10]
3',4',7-Trihydroxyisoflavone	$C_{15}H_{10}O_5$	JX	[1]
Kaempferol-3-O-rutinoside	$C_{27}H_{30}O_{15}$	HH, CG	[15, 25]
Luteolin-7-O-β-D-glucoside	$C_{21}H_{20}O_{11}$	CG	[15]
Kaempferol-3-O-β-D-glucoside	$C_{21}H_{20}O_{11}$	MHZ	[14]
Kaempferol-3-O-rutinoside	$C_{27}H_{30}O_{15}$	HZ	[3]
Kaempferol-5-0-arabinoside	$C_{20}H_{18}O_{10}$	CG	[8]
Quercetin-3-0-rhamnoside	$C_{21}H_{20}O_{11}$,	CG	[8]

Compounds	Formula	Source	Ref
Myricetin-3-0-rhamnoside	$C_{21}H_{20}O_{12}$	CG	[8]
Eriodictyol-7-O-β-D-glucoside	$C_{21}H_{22}O_{11}$	MHZ	[14]
Diosmetin-7-O-β-D-glucoside	$C_{22}H_{22}O_{11}$	MHZ	[14]
5,7-Dihydroxy-4'-methoxyflavone	$C_{16}H_{12}O_{5}$	HH	[40]
4′,5-Dihydroxyflavanone-6,7-di-O-β-D-glucoside	$C_{27}H_{32}O_{16}$	HH	[40]
Quercetin-7-O-β-D-glucoside	$C_{21}H_{20}O_{12}$	CG	[22]
Quercetin-3-O-β-D-glucoside	$C_{21}H_{20}O_{12}$	CG	[22]
Organic acids			
Wilsonic acid	$C_{10}H_{7}NO_{3}$	JX	[21]
Gallic acid	$C_{7}H_{6}O_{5}$	GZ, MHZ, CG, YGZ, DX, DS, HZ	[3, 16, 41-44]
Benzoic acid	$C_{7}H_{6}O_{2}$	HZ	[45]
3,4-Dihydroxybenzoic acid	$C_{7}H_{6}O_{4}$	CG,	[28]
Syringic acid	$C_{7}H_{6}O_{3}$	CG, HZ, DK	[28, 34, 36]
4-Hydroxybenzoic acid	$C_{7}H_{6}O_{3}$	CG	[28]
Gentisica cid	$C_{7}H_{6}O_{5}$	DK	[34]
Pyrogallol	$C_{6}H_{6}O_{3}$	HZ	[46]
Vanillic acid	$C_{8}H_{8}O_{3}$	DK, AW, MHZ	[7, 28, 34, 37]
Mucic acid	$C_{6}H_{10}O_{8}$	YGZ	[38]
Hydroxybenzoic acid	$C_{7}H_{6}O_{3}$	GZ, HH	[16, 26]
Salicylic acid	$C_{7}H_{6}O_{3}$	JX, GZ	[6, 16]
Methylsalicylate	$C_{8}H_{8}O_{3}$	DX	[47]

Compounds	Formula	Source	Ref
Ethylgallate	$C_{10}H_{12}O_4$	GZ, MHZ	[16, 43]
1,5-Dicaffeoylquinic acid	$C_{25}H_{22}O_{12}$	AW	[48]
Malic acid	$C_4H_6O_5$	YGZ	[38]
Docosahexaenoic acid	$C_{22}H_{32}O_2$	JX	[49]
Oleic acid	$C_{18}H_{34}O_2$	JX, CX, ZHX, TX	[30, 49-51]
Nonanoic acid	$C_9H_{18}O_2$	MHZ	[7]
Arachidonic acid	$C_{20}H_{32}O_2$	JX	[49]
Nervonic acid	$C_{24}H_{46}O_2$	CG	[52]
α-Linolenic acid	$C_{18}H_{30}O_2$	JX, YGZ, ZHX	[49, 51, 53]
Erucic acid	$C_{22}H_{42}O_2$	JX	[49]
Linoleic acid	$C_{18}H_{32}O_2$	CG, ZHX, YGZ, RDK	[50, 52, 54, 55]
Myristic acid	$C_{14}H_{28}O_2$	RDK,	[55]
3-Methyldecanoic acid	$C_{11}H_{22}O_2$	RDK	[55]
Palmitic acid	$C_{16}H_{32}O_2$	ZHX, JX, CX, TX	[50, 56-58]
Ethylpalmitate	$C_{18}H_{36}O_2$	CX	[30]
Stearic acid	$C_{18}H_{36}O_2$	CX, GZ, ZHX	[16, 51, 59]
Lauric acid	$C_{12}H_{24}O_2$	RDK	[55]
Methylmyristate	$C_{15}H_{30}O_2$	RDK	[55]
4,4-Dimethylpimelic acid	$C_9H_{16}O_4$	HH	[60]
Lauric acid	$C_{12}H_{24}O_2$	CX	[30]
Tannins			
ProcyanidinB2	$C_{30}H_{26}O_{12}$	CG	[35]

Compounds	Formula	Source	Ref
methylneochebulanin	$C_{37}H_{32}O_{18}$	MHZ	[61]
ProcyanidinB-typetetramer	$C_{60}H_{50}O_{24}$	CG	[28]
Chebulanin	$C_{41}H_{32}O_{26}$	HZ	[62]
Corilagin	$C_{34}H_{24}O_{22}$	HZ	[62]
Neochebulanin	$C_{41}H_{30}O_{27}$	HZ	[62]
TerminalinA	$C_{34}H_{24}O_{22}$	HZ	[62]
Breviaranoicacid	$C_{14}H_{12}O_{5}$	HZ	[62]
meta-Digallicacid	$C_{14}H_{10}O_{9}$	HZ	[62]
tercatain	$C_{34}H_{24}O_{22}$	HZ	[62]
geminD	$C_{41}H_{30}O_{27}$	HZ	[62]
tellimagrandinI	$C_{34}H_{24}O_{22}$	HZ	[62]
punicacorteinC	$C_{48}H_{28}O_{30}$	HZ	[62]
punicacorteinD	$C_{48}H_{28}O_{30}$	HZ	[62]
methylneochebulagate	$C_{37}H_{32}O_{18}$	HZ	[62]
methylneochebulinate	$C_{37}H_{32}O_{18}$	HZ	[62]
Neochebulinicacid	$C_{34}H_{24}O_{22}$	HZ	[45]
4-O-Galloyl-(-)-shikimicacid	$C_{14}H_{14}O_{10}$	HZ	[62]
5-O-Galloyl-(-)-shikimicacid	$C_{14}H_{14}O_{10}$	HZ	[62]
1,3-Di-O-galloyl-β-D-glucopyranoside	$C_{20}H_{20}O_{14}$	HZ	[62]
1,2,3,6-Tetra-O-galloyl-β-D-glucopyranoside	$C_{34}H_{28}O_{22}$	HZ	[62]
1,2-Di-O-galloyl-6-O-cinnamoyl-β-D-glucopyranoside	$C_{29}H_{26}O_{14}$	HZ	[62]

Compounds	Formula	Source	Ref
1,6-Di-O-galloyl-2-O-cinnamoyl-β-D-glucopyranoside	$C_{29}H_{26}O_{14}$	HZ	[62]
1,2,3-Tri-O-galloyl-6-O-cinnamoyl-β-D-glucopyranoside	$C_{37}H_{30}O_{18}$	HZ	[62]
Geraniin	$C_{41}H_{28}O_{27}$	YGZ, HZ	[63, 64]
Chlorinatedanthocyanin	$C_{15}H_{11}ClO_{6}$	EC	[24]
Chebulic acid	$C_{41}H_{30}O_{27}$	YGZ, MHZ	[43, 64]
Proto-chebulinic acid	$C_{34}H_{24}O_{22}$	YGZ	[64]
Isomallotusinin	$C_{34}H_{24}O_{22}$	YGZ	[64]
Methylchebulagate	$C_{41}H_{30}O_{27}$	YGZ	[64]
Neochebulagic acid	$C_{41}H_{30}O_{27}$	YGZ	[64]
Punicafolin	$C_{48}H_{28}O_{30}$	YGZ	[64]
Mallonin	$C_{34}H_{24}O_{22}$	YGZ	[64]
Punicalagin	$C_{48}H_{28}O_{30}$	HZ	[63]
Vitexin	$C_{10}H_{8}O_{4}$	HZ	[63]
Ellagic acid	$C_{14}H_{6}O_{8}$	GZ, HZ, YGZ	[16, 36, 38]
3,3-Dimethylellagic acid	$C_{16}H_{10}O_{8}$	GZ	[16]
Ellagicacidglycoside	$C_{20}H_{16}O_{13}$	YGZ	[38]
Galloyl-HHDP-glucose	$C_{27}H_{22}O_{18}$	YGZ	[38]
Gallicacid-3,6-hexahydroxydiphenoyl-glucose	$C_{27}H_{22}O_{18}$	YGZ	[38]
putranjivainA	$C_{34}H_{24}O_{22}$	YGZ	[38]
elaeocarpusin	$C_{41}H_{28}O_{26}$	YGZ	[38]
Chebulagic acid	$C_{41}H_{30}O_{27}$	YGZ	[38]

**Compounds**	Formula	**Source**	**Ref**
Neochebulagic acid	$C_{41}H_{30}O_{26}$	YGZ	[38]
chebulanin	$C_{34}H_{24}O_{22}$	YGZ	[38]
EschweilenolC	$C_{34}H_{24}O_{22}$	HZ	[62]
PhyllanemblininE	$C_{34}H_{24}O_{22}$	HZ	[62]
1-O-Methylneochebulic acid	$C_{35}H_{26}O_{22}$	HZ	[62]
Dimethylneochebulic acid	$C_{36}H_{28}O_{22}$	HZ	[62]
6-O-Methylneochebulic acid	$C_{35}H_{26}O_{22}$	HZ	[62]
Neochebulic acid	$C_{34}H_{24}O_{22}$	HZ	[62]
Chebulinic acid	$C_{41}H_{32}O_{27}$	HZ	[62]
7′-O-methylchebulate	$C_{15}H_{14}O_{11}$	HZ	[62]
6′-O-methylchebulate	$C_{15}H_{14}O_{11}$	HZ	[62]
OtherNonvolatileCompounds			
1H-Cyclopropa[a]naphthalene	$C_{11}H_{8}$	RDK	[65]
Buprofezin	$C_{16}H_{19}N_{3}O_{2}S$	JX	[49]
Cyclopentylisothiocyanate	$C_{6}H_{9}NS$	RDK	[66]
Acetyl-β-boswellic acid	$C_{32}H_{50}O_{4}$	RX	[67]
Octanenitrile	$C_{8}H_{15}N$	MHZ	[68]
Aquilegiolide	$C_{19}H_{28}O_{4}$	CX	[69]
proline	$C_{5}H_{9}NO_{2}$	GZ	[16]
phenylalanine	$C_{9}H_{11}NO_{2}$	GZ	[16]
scopoletin	$C_{10}H_{8}O_{4}$	GZ	[16]
bilirubin	$C_{33}H_{36}N_{4}O_{6}$	NH	[70]
Kirenol	$C_{30}H_{50}O_{2}$	DX	[13]

**Compounds**	Formula	**Source**	**Ref**
taurine	$C_2H_7NO_3S$	NH	[70]
γ-butyrolactone	$C_4H_6O_2$	CG, AW	[48, 71]
Neoisolongifolan	$C_{15}H_{24}O$	RDK	[65]
Tributylphosphate	$C_{12}H_{27}O_4P$	JX	[49]
MalabariconeC	$C_{24}H_{38}O_4$	RDK	[72]
6-Hydroxyindolelacticacid	$C_{11}H_{11}NO_4$	GZ	[16]
Tyrosine	$C_9H_{11}NO_3$	GZ	[16]
Bileacids	$C_{24}H_{40}O_5$	NH	[70]
VitaminE	$C_{29}H_{50}O_2$	HZ, ZHX	[46, 51]
Ursolicacid	$C_{30}H_{48}O_3$	DX	[13]
Lutein	$C_{40}H_{56}O_2$	ZHX	[51]
β-Carotene	$C_{40}H_{56}$	ZHX	[51]
Glycocholic acid	$C_{26}H_{43}NO_5$	NH	[73]
Taurochenodeoxycholic acid	$C_{26}H_{45}NO_6S$	NH	[73]
Glycochenodeoxycholic acid	$C_{26}H_{43}NO_5$	NH	[73]
Ursodeoxycholic acid	$C_{24}H_{40}O_4$	NH	[73]
Hyodeoxycholic acid	$C_{24}H_{40}O_4$	NH	[73]
Glycodeoxycholic acid	$C_{26}H_{43}NO_5$	NH	[73]
Deoxycholic acid	$C_{24}H_{40}O_4$	NH	[73]
β-Sitosterol	$C_{29}H_{50}O$	DK, ZHX, JX, CG, YGZ	[22, 34, 54, 56, 58, 69]
Arjunolic acid	$C_{14}H_{24}O$	HZ	[74]
Arjunetin	$C_{15}H_{24}O$	HZ	[74]

Compounds	Formula	Source	Ref
Ursolic acid	$C_{15}H_{24}O$	GZ	[16]
Chebulic acid	$C_{18}H_{30}O$	YGZ, HZ	[3, 64, 75]
Lithocholic acid	$C_{24}H_{40}O_3$	NH	[73]
DiformylquinoneA	$C_{20}H_{18}O_6$	AW	[48]
Taurocholic acid	$C_{26}H_{45}NO_7S$	NH	[73]
Uridine	$C_9H_{12}N_2O_6$	AW	[48]
Monoterpenes			
Dehydrolinalool	$C_{10}H_{18}O$	JX	[49]
Citral	$C_{10}H_{16}O$	CX	[29]
Citronellol	$C_{10}H_{20}O$	CX, RDK	[29, 66]
Thymol	$C_{10}H_{14}O$	RDK	[29]
Citronellal	$C_{10}H_{18}O$	RX	[76]
β-Ionone	$C_{13}H_{20}O$	TX	[77]
Teresantalol	$C_{10}H_{18}O$	TX	[77]
Dihydroconiferylalcohol	$C_{10}H_{14}O_2$	TX	[78]
Methoxycitronellal	$C_{11}H_{22}O_2$	MHZ	[68]
Menthol	$C_{10}H_{20}O$	RX	[76]
β-Ocimene	$C_{10}H_{16}$	CX	[29]
cis-Geranylacetate	$C_{12}H_{20}O_2$	RDK	[79]
2,6-Dimethyl-2,6-octadiene	$C_{10}H_{18}$	RDK	[79]
Nerolidol	$C_{15}H_{26}O$	JX, CX	[56, 80]
Geraniol	$C_{10}H_{18}O$	DK, RX	[34, 76]
2,6-Dimethyl-2,4,6-octatriene	$C_{10}H_{16}$	CX	[29]

**Compounds**	Formula	**Source**	**Ref**
Nerol	$C_{10}H_{18}O$	CX	[29]
(Z)-3,7-Dimethyl-2,6-octadienal	$C_{10}H_{16}O$	CG	[81]
α-Terpinene	$C_{10}H_{16}$	CX, RDK	[29, 55, 79]
cis-β-Terpineol	$C_{10}H_{18}O$	RDK	[79]
Terpinen-4-ylacetate	$C_{12}H_{20}O_2$	RDK	[79]
(1R,2S,5R) -5-Methyl-2- (propan-2-yl) cyclohex-2-en-1-ol	$C_{10}H_{18}O$	RDK	[79]
(1R,4S) -1-Methyl-4-(propan-2-yl) cyclohex-2-en-1-ol	$C_{10}H_{18}O$	RDK	[79]
2-Butyl-3-methyl-5- (2-methylprop-2-en-1-yl) cyclohexanone	$C_{15}H_{26}O$	JX	[49]
Damascenone	$C_{13}H_{18}O$	JX	[49]
Safranal	$C_{10}H_{14}O$	JX	[49]
Carvomenthol	$C_{10}H_{16}O$	CX	[29]
D-Limonene	$C_{10}H_{16}$	CX, RDK, AW, RX, TX	[29, 48, 62, 79, 82]
Carvacrol	$C_{10}H_{14}O$	RDK	[83]
p-Cymene	$C_{10}H_{14}$	RX, TX	[62, 82]
Anisylalcohol	$C_8H_{10}O_2$	RX	[62]
Anisylacetate	$C_{10}H_{12}O_3$	RX	[62]
Menthylacetate	$C_{12}H_{22}O_2$	RX	[62]
Isomenthone	$C_{10}H_{18}O$	RX	[62]
Geranylacetate	$C_{12}H_{20}O_2$	RX	[62]
Bornylacetate	$C_{12}H_{20}O_2$	RX	[62]

Compounds	Formula	Source	Ref
Menthone	$C_{10}H_{18}O$	RX	[62]
Perillylalcohol	$C_{10}H_{16}O$	RDK	[83]
D-Carvone	$C_{10}H_{14}O$	CX	[29]
α-Phellandrene	$C_{10}H_{16}$	RDK, ZHX, CG	[79]
1-Terpinen-4-ol	$C_{10}H_{18}O$	RDK, CG	[79, 81]
α-Terpinylacetate	$C_{12}H_{20}O_2$	RDK	[79]
1-Methyl-5- (propan-2-yl) cyclohex-1-ene	$C_{10}H_{18}$	RDK	[79]
1-Methylcyclodec-1-ene	$C_{11}H_{20}$	RDK	[79]
α-Terpineol	$C_{10}H_{18}O$	DK, JX, CG	[34, 49, 81]
Perillaldehyde	$C_{10}H_{14}O$	CX	[29]
β-Phellandrene	$C_{10}H_{16}$	CX	[29]
Dihydrocarveol	$C_{10}H_{18}O$	CX	[29]
Terpinene	$C_{10}H_{16}$	CX, RDK, DX	[29, 47, 79]
1,4-Dimethyl-4-acetylcyclohex-1-ene	$C_{10}H_{16}O$	CX	[29]
(4R,6S)-4-Hydroxy-3-methyl-6-(propan-2-yl)cyclohex-2-en-1-one	$C_{10}H_{16}O_2$	DS	[84]
α-pinene	$C_{10}H_{16}$	RDK, CG, AW, RX	[55]
Camphene	$C_{10}H_{16}$	CG, RDK, CX, TX	[55, 59, 71, 81-83]
Borneol	$C_{10}H_{18}O$	RDK	[55]
1,8-Cineole	$C_{10}H_{18}O$	RDK, CG, DS	[29, 81, 84]
Dihydroactinidiolide	$C_{11}H_{18}O_2$	JX	[49]
Fenchone	$C_{10}H_{16}O$	RDK	[29]

**Compounds**	Formula	**Source**	**Ref**
Isoborneol	$C_{10}H_{18}O$	DS	[84]
endo-Bornylacetate	$C_{12}H_{20}O_2$	RDK, DS	[83, 84]
Isoborneol	$C_{10}H_{18}O$	RDK	[85]
trans-4-Methoxythujone	$C_{11}H_{20}O$	RDK	[85]
Isobornylacetate	$C_{12}H_{20}O_2$	RDK	[66]
Isotsaokoin	$C_{15}H_{22}O$	CG	[86]
Sabinene	$C_{10}H_{16}$	RDK, ZHX	[29, 58, 79, 83]
β-Pinene	$C_{10}H_{16}$	RDK, CG, ZHX, DS, DX	[6, 55, 58, 79, 81, 87, 88]
Safrole	$C_{10}H_{10}O_2$	RDK	[85]
6-Amino-3,3-dimethylindoline	$C_{10}H_{14}N_2$	RDK	[29]
2-Camphanone	$C_{10}H_{16}O$	RDK	[29]
Camphor	$C_{10}H_{16}O$	DS	[84]
endo-Borneol	$C_{10}H_{18}O$	DS	[84]
cis-4-Methoxythujane	$C_{11}H_{20}O$	RDK	[85]
α-Thujene	$C_{10}H_{16}$	RDK	[79, 83]
Fenchol	$C_{10}H_{18}O$	RDK	[66]
Sesquiterpenes			
Bergamotene	$C_{15}H_{24}$	RDK	[66]
(E)-β-Farnesene	$C_{15}H_{24}$	RDK	[55]
Bisabolene	$C_{15}H_{24}$	RDK	[55]

Compounds	Formula	Source	Ref
Guaiol	$C_{15}H_{26}O$	RDK, DS	[66, 84, 85]
Hinesol	$C_{15}H_{26}O$	CX	[80]
Aromadendrene	$C_{15}H_{24}$	TX	[89]
safflomegastigside	$C_{35}H_{46}O_8$	HH	[90]
β-Costol	$C_{15}H_{26}O$	TX	[89]
Caryophylleneoxide	$C_{15}H_{24}O$	TX	[89]
(9S,10E) -9-hydroxy-α-santalol	$C_{15}H_{26}O_2$	TX	[91]
(10R,11S) -10,11-dihydroxy-α-santalol	$C_{15}H_{26}O_3$	TX	[91]
(10E) -12-hydroxy-a-santalicacid	$C_{15}H_{22}O_3$	TX	[91]
α-Funebrene	$C_{15}H_{24}$	TX	[92]
α-Cedrene	$C_{15}H_{24}$	TX	[92]
α-Santalene	$C_{15}H_{24}$	TX	[92]
α-trans-bergamotene	$C_{15}H_{24}$	TX	[92]
Sesquisabinene	$C_{15}H_{24}$	TX	[92]
β-Acoradiene	$C_{15}H_{24}$	TX	[92]
Ar-curcumene	$C_{15}H_{22}$	TX	[92]
β-Bisabolene	$C_{15}H_{24}$	TX	[92]
Bulnesol	$C_{15}H_{26}O$	TX	[92]
Caryophylleneoxide	$C_{15}H_{24}O$	ZHX, JX, DX	[47, 58, 87, 93]
Caryophylleneacetate	$C_{17}H_{28}O_2$	DX	[94]
(2E,6E,10E)-6,11-Dimethyldodeca-2,6,10-trien-1-ol	$C_{14}H_{24}O$	JX	[93]
Santalol	$C_{15}H_{24}O$	JX	[56]

Compounds	Formula	Source	Ref
Santalol	$C_{15}H_{24}O$	RDK, JX, CX, TX	[29, 49, 59]
Farnesylacetone	$C_{18}H_{30}O$	JX	[49]
Patchoulol	$C_{15}H_{26}O$	RDK	[29]
α-Bulnesene	$C_{15}H_{24}$	RDK	[29]
α-Cedrene	$C_{15}H_{24}$	RDK, RX	[29, 76]
Elemene	$C_{15}H_{24}$	JX, CX, RDK, DS, RX	[55, 59, 62, 84, 95]
GermacreneD	$C_{15}H_{24}$	RDK	[66]
Caryophyllene	$C_{15}H_{24}$	RDK, CX, RX	[59, 66, 76, 79, 83, 95]
Amyrin	$C_{30}H_{50}O$	RX	[67, 76]
Eudesmol	$C_{15}H_{26}O$	RDK, ZHX	[29, 58]
Santalene	$C_{15}H_{24}$	JX	[93]
(10E) -7,11-Dimethyldodec-10-en-1-ol	$C_{14}H_{28}O$	JX	[93]
Selinenes	$C_{15}H_{24}$	DS, JX, DX	[47, 56, 84]
β-Curcumene	$C_{15}H_{22}$	JX, DS	[49, 84]
Cedrol	$C_{15}H_{26}O$	RDK, JX	[29, 49]
Amyrene	$C_{15}H_{24}$	RDK, CG	[29, 71, 85]
Agarospirol	$C_{15}H_{24}O$	RDK, CX	[29, 80]
α-Farnesene	$C_{15}H_{24}$	RDK, CX, ZHX	[29, 58, 59, 66]
β-Humulene	$C_{15}H_{24}$	RDK, CG, ZHX, DX, CX	[6, 29, 58, 71, 95]
(+)-GermacreneD	$C_{15}H_{24}$	RDK	[29]

**Compounds**	Formula	**Source**	**Ref**
β-Cedrene	$C_{15}H_{24}$	DS	[84]
trans-Caryophyllene	$C_{15}H_{24}$	RDK, DS, DX	[47, 84, 85]
Farnesol	$C_{15}H_{26}O$	DS	[84]
Farnesal	$C_{15}H_{24}O$	DS	[84]
Bisabolene	$C_{15}H_{24}$	RDK	[66]
Atractylol	$C_{15}H_{26}O$	CX	[57]
Agarospirol	$C_{15}H_{24}O$	CX	[30]
α-Costene	$C_{15}H_{24}$	RX	[76]
Cubebene	$C_{15}H_{24}$	RDK	[83]
Ledol	$C_{15}H_{26}O$	ZHX	[58]
Bourbonene	$C_{15}H_{24}$	CG	[71]
Cubebol	$C_{15}H_{26}O$	DK	[34]
Ferulasin	$C_{20}H_{24}O_4$	AW	[96]
α-Guaiene	$C_{15}H_{24}$	DS, CX, RX	[30, 76, 84]
β-Guaiene	$C_{15}H_{24}$	DS	[84]
γ-Guaiene	$C_{15}H_{24}$	RX	[76]
β-Bisabolene	$C_{15}H_{24}$	DS	[84]
γ-Muurolene	$C_{15}H_{24}$	DS, RDK	[66, 84]
Longifolene	$C_{15}H_{24}$	RDK, CX	[59, 85]
(±)-δ-Cadinene	$C_{15}H_{24}$	RDK	[66]
Cadinol	$C_{15}H_{26}O$	CX	[95]
Cadinene	$C_{15}H_{24}$	DX	[47]
GermacreneD	$C_{15}H_{24}$	DX	[47]

**Compounds**	Formula	**Source**	**Ref**
Muurolene	$C_{15}H_{24}$	DX	[47]
Baimuxinicacid	$C_{15}H_{24}$	CX	[57]
Eudesmol	$C_{15}H_{26}O$	CX	[59]
Caryophyllenol	$C_{15}H_{24}$	ZHX	[58]
Farnesol	$C_{35}H_{46}O_8$	DK, DX	[6, 34]
α-Calacorene	$C_{15}H_{26}O$	DK	[34]
Globulol	$C_{15}H_{24}O$	DK	[34]
GermacreneD	$C_{15}H_{26}O_2$	RDK	[83]
Calamenene	$C_{15}H_{26}O_3$	RDK	[65]
Bicyclogermacrene	$C_{15}H_{22}O_3$	RDK	[29]
γ-Terpinene	$C_{15}H_{24}$	RX	[76]
Ditepenoids			
Retinal	$C_{20}H_{28}O$	JX	[49]
Anethole	$C_{20}H_{34}O_2$	RX	[97]
dilospirane	$C_{20}H_{32}O$	RX	[97]
dictyotin	$C_{20}H_{30}O_2$	RX	[97]
Cembranol	$C_{20}H_{34}O$	RX	[97]
euphraticanoid	$C_{15}H_{26}O$	RX	[97]
Phenylpropanoids			
Elemicin	$C_{12}H_{14}O_3$	RDK	[66]
Coumarin	$C_9H_6O_2$	JX	[56]
Asarone	$C_{10}H_{12}O_2$	ZHX, JX	[56, 58]
Ageratochromene	$C_{15}H_{14}O_6$	JX	[56]

Compounds	Formula	Source	Ref
β-Gurjunene	$C_{15}H_{24}$	JX, RX	[56, 76]
1,3-Diphenyl-2-buten-1-one	$C_{16}H_{14}O$	JX	[49]
α-Cubebene	$C_{15}H_{24}$	RDK	[29, 85]
5-O-Caffeoylquinic acid	$C_{16}H_{18}O_9$	DK	[34]
α-Methylcinnamaldehyde	$C_{10}H_{10}O$	CG, CX	[59, 81]
Cinnamaldehyde	C_9H_8O	RX	[76]
Ferulic acid	$C_{10}H_{10}O_4$	DK, DX, CG, AW, MHZ	[6, 7, 28, 34, 37]
Caffeic acid	$C_9H_8O_4$	DK, DX, CG, AW, YGZ	[6, 28, 34, 37, 38]
p-Coumaric acid	$C_9H_8O_3$	HH	[26]
Chlorogenic acid	$C_{16}H_{18}O_9$	HH, AW	[25, 37]
4-Hydroxycinnamic acid	$C_9H_8O_3$	DK	[34]
Eugenol	$C_{10}H_{12}O_2$	RDK, DX, RX	[6, 55, 62]
Methylisoeugenol	$C_{11}H_{14}O_2$	RDK	[66]
Protocatechuic acid	$C_7H_6O_4$	DK, GZ, MHZ, HZ	[3, 16, 34, 42]
Isoeugenol	$C_{10}H_{12}O_2$	DX	[47]
Methyleugenol	$C_{11}H_{14}O_2$	DX	[94]
7-Hydroxycoumarin	$C_9H_6O_3$	AW	[7, 48]
p-Coumaric acid	$C_9H_8O_3$	DS	[44]
7,8-erythro-4,9,9'-trihydroxy-3,3'-dimethoxy-8.O.4'-neolignan	$C_{20}H_{24}O_8$	TX	[98]

**Compounds**	Formula	**Source**	**Ref**
7,8-threo-4,9,9'-trihydroxy-3,3'-dimethoxy-8.O.4'-neolignan	$C_{20}H_{24}O_8$	TX	[98]
(7S,8S)-3-methoxy-3',7-epoxy-8,4'-oxyneoligna-4,9,9'-triol	$C_{20}H_{24}O_7$	TX	[98]
(7S,8R,8'R)-lyoniresinol	$C_{20}H_{24}O_8$	TX	[98]
secoisolariciresinol	$C_{20}H_{26}O_6$	TX	[98]
Aromatic compounds			
Phenylacetylene	C_8H_6	CX	[59]
p-Cymene	$C_{10}H_{14}$	RDK	[29, 79]
Anthracene	$C_{14}H_{10}$	CX	[59]
o-Isopropylbenzene	C_9H_{12}	CG	[71]
Styrene	C_8H_8	CX	[59]
Isopropenyltoluene	$C_{10}H_{12}$	RDK	[29]
p-Xylene	C_8H_{10}	CG	[71]
Toluene	C_7H_8	CG	[71]
5-Isopropyl-2-methylphenol	$C_{10}H_{14}O$	RDK	[55]
Benzylalcohol	C_7H_8O	JX	[49]
Phenethylalcohol	$C_8H_{10}O$	JX	[49]
4-tert-Butylphenol	$C_{10}H_{14}O$	RDK, ZHX	[29, 58]
Benzylbenzoate	$C_{14}H_{12}O_2$	DX	[47]
Anisole	C_7H_8O	CX	[59]
4-Allylanisole	$C_{10}H_{12}O$	CG	[71]
p-Anisaldehyde	$C_8H_8O_2$	MHZ	[68]

Compounds	Formula	Source	Ref
m-Cresol	C_7H_8O	CX	[59]
o-tert-Butylphenol	$C_{10}H_{14}O$	CX	[59]
p-Pentylanisole	$C_{12}H_{18}O$	RDK	[55]
4-Vinyl-2-methoxyphenol	$C_9H_{10}O_2$	JX	[49]
2,4-Di-tert-butylphenol	$C_{14}H_{22}O$	JX	[49]
Tetramethyl-1,4-benzoquinone	$C_{10}H_{14}O_2$	RDK	[29]
1,7-Bis(4-hydroxyphenyl)-3,5-heptanediol	$C_{19}H_{24}O_4$	CG	[86]
Phenol	C_6H_6O	CX	[59]
Catechol	$C_6H_6O_2$	CX, CG	[59]
Homovanillylalcohol	$C_9H_{12}O_2$	CX	[59]
Isovanillin	$C_8H_8O_3$	RDK	[55]
p-Allylphenol	$C_9H_{10}O$	DX	[94]
Salicylaldehyde	$C_7H_6O_2$	JX	[56]
Cuminaldehyde	$C_{10}H_{12}O$	RDK	[29]
Benzylacetone	$C_{10}H_{12}O$	RDK	[29]
1-Indanone	C_9H_8O	CX	[59]
p-Anisaldehyde	$C_8H_8O_2$	RDK	[29]
2,3-Dihydroxybenzaldehyde	$C_7H_6O_3$	CX	[59]
Anisaldehyde	$C_8H_8O_2$	CX	[59]
Ethylvanillate	$C_{10}H_{12}O_3$	CX	[59]
Ethylbenzoate	$C_9H_{10}O_2$	CX	[30]
Benzaldehyde	C_7H_6O	CG, DX, JX	[6, 56, 71]
3,4-Dihydroxybenzaldehyde	$C_7H_6O_3$	CG	[28]

Compounds	Formula	Source	Ref
Protocatechualdehyde	$C_7H_6O_3$	GZ	[16]
2-Phenylbutyraldehyde	$C_{10}H_{12}O$	CG, MHZ	[68, 81]
Vanillin	$C_8H_8O_3$	CX, YGZ	[54, 59]
3-Hydroxybenzaldehyde	$C_7H_6O_2$	CX	[59]
9-Fluorenone	$C_{13}H_8O$	CX	[59]
Benzil	C_7H_6O	CX	[59]
p-Isopropylbenzoic acid	$C_{10}H_{12}O_2$	DS	[84]
Methylphenylacetate	$C_9H_{10}O_2$	RDK	[29]
5-Methylfurfural	$C_6H_6O_2$	CX	[59]
2-Acetylfuran	$C_6H_6O_2$	DS	[84]
2,3-Dihydrobenzofuran	C_8H_8O	CX	[59]
5-Hydroxymethylfurfural	$C_6H_6O_3$	CX, GZ, AW	[16, 48, 59]
2,6-Dihydroxy-4-methylquinoline	$C_{10}H_9NO_2$	DS	[84]
2,4,6-Trimethylpyridine	$C_8H_{11}N$	CX	[59]
Furfural	$C_5H_4O_2$	RDK, CG	[29, 71]
Cyclooctane	C_8H_{16}	CG	[81]
Cycloheptene	C_7H_{12}	CG	[81]
Heneicosane	$C_{21}H_{44}$	CX	[59]
Vinylcyclohexane	C_8H_{14}	MHZ	[68]
Hexadecane	$C_{16}H_{34}$	DX	[47]
Dipentene	$C_{10}H_{16}$	DX	[94]
Cyclododecene	$C_{12}H_{22}$	CG	[81]
Tridecane	$C_{13}H_{28}$	CG, DX	[47, 71]

Compounds	Formula	Source	Ref
2-Methyldecane	$C_{11}H_{24}$	MHZ	[68]
Tetradecane	$C_{14}H_{30}$	CG	[71]
Cyclopentane	C_5H_{10}	CX	[59]
10-Dodecyn-1-ol	$C_{12}H_{22}O$	JX	[93]
2-Hexadecanol	$C_{16}H_{34}O$	JX	[49]
Isophytol	$C_{20}H_{40}O$	JX	[49]
Phytol	$C_{20}H_{40}O$	JX, YGZ	[49, 54]
2,3-Butanediol	$C_4H_{10}O_2$	RDK	[29]
2-Cyclohexenol	$C_6H_{10}O$	RDK	[85]
2-Ethylhexanol	$C_8H_{18}O$	RDK	[29]
(E)-2-Decenal	$C_{10}H_{18}O$	MHZ	[68]
3,7,11-Trimethyldodecanol	$C_{15}H_{32}O$	JX	[49]
3,7-Dimethyl-1,7-octanediol	$C_{10}H_{22}O_2$	JX	[49]
1-Heptanol	$C_7H_{16}O$	DS	[84]
1-Hexanol	$C_6H_{14}O$	RDK	[29]
2-Hexanol	$C_6H_{14}O$	CG	[71]
1-Hexacosanol	$C_{26}H_{54}O$	AW	[48]
Hexanal	$C_6H_{12}O$	RDK, MHZ	[29, 68]
2-Nonanone	$C_9H_{18}O$	CG	[71]
Acetaldehyde	C_2H_4O	JX	[49]
Tetradecanal	$C_{14}H_{28}O$	TX	[50]
Hexadecanal	$C_{16}H_{32}O$	TX	[50]
Octadecanal	$C_{18}H_{36}O$	TX	[50]

Compounds	Formula	Source	Ref
(E)-2-Heptenal	$C_7H_{12}O$	MHZ	[68]
(E, E)-2,4-Nonadienal	$C_9H_{14}O$	MHZ	[68]
Octanal	$C_8H_{16}O$	RDK, CG, MHZ	[29, 68, 71, 81]
(E)-2-Dodecenal	$C_{12}H_{22}O$	DS	[84]
(E, E)-2,4-Octadienal	$C_8H_{12}O$	DS	[84]
Phytone	$C_{18}H_{36}O$	JX	[49]
Nonanal	$C_9H_{18}O$	RDK, MHZ	[29, 68]
Decanal	$C_{10}H_{20}O$	CG	[81]
2-Ethyl-1-hexanol	$C_{18}H_{18}O$	MHZ	[68]
1-Octanol	$C_8H_{18}O$	MHZ	[68]
6-Methyl-5-hepten-2-one	$C_8H_{14}O$	RDK	[29]
(E)-2-Octenal	$C_8H_{14}O$	DS	[84]
(E)-2-Hexenal	$C_6H_{10}O$	DS	[84]
Quinic acid	$C_7H_{12}O_6$	DK, GZ, MHZ	[16, 34, 99]
Succinic acid	$C_4H_6O_4$	GZ	[16]
Ethylacetate	$C_4H_8O_2$	RDK, CG	[29, 71]
Methylbutyrate	$C_5H_{10}O_2$	CG	[71]
(E)-2-Decenal	$C_{10}H_{18}O$	DS	[84]
Hexylacetate	$C_8H_{16}O_2$	RDK	[29]
Methylacetate	$C_3H_6O_2$	CG	[71]
Dodec-2-en-1-ylacetate	$C_{14}H_{26}O_2$	DS	[84]
11-Dodecyn-1-ylacetate	$C_{14}H_{22}O_2$	JX	[93]

Compounds	Formula	Source	Ref
Mucic acid 6-methyl ester 2-O-gallate	$C_{14}H_{16}O_{12}$	YGZ	[100]
(E)-1-Propenylsec-butyldisulfide	$C_7H_{14}S_2$	AW	[48]
(Z)-1-Propenylsec-butyldisulfide	$C_7H_{14}S_2$	AW	[48]
1- (1-Propenylthio) propylmethyldisulfide	$C_7H_{14}S_3$	AW	[48]
Bis (1-methylpropyl) disulfide	$C_8H_{18}S_2$	AW	[48]
Bis[1- (methylthio) propyl] disulfide	$C_8H_{18}S_4$	AW	[48]
Bis[1- (methylthio) ethyl] disulfide	$C_6H_{14}S_4$	AW	[48]
Dimethyltrisulfide	$C_2H_6S_3$	AW	[48]
Butylmethyldisulfide	$C_5H_{12}S_2$	AW	[48]

Abbreviations: RDK: Roudoukou; JX: Jiangxiang; CX: Chenxiang; GZ: Guangzao; HH: Honghua; ZHX: Zanghuixiang; DX: Dingxiang; DS: Dasuan; DK: Doukou; AW: Awei; CG: Caoguo; HZ: Hezi; RX: Ruxiang; MHZ: Maohezi; EC: Ercha; YGZ: Yuganzi; TX: Tanxiang; NH: Niuhuang.

References

1. Sun, K.; Su, C. N.; Li, W. J.; Gong, Z.; Sha, C. J., Quality markers based on phytochemical analysis and anti-inflammatory screening: An integrated strategy for the quality control of Dalbergia odorifera by UHPLC-Q-Orbitrap HRMS. *Phytomedicine* **2021,** 84, 153511-153511.http://doi.org/10.1016/J.PHYMED.2021.153511.
2. RongHua, L.; QianQian, Y.; XiaoWei, M.; Qing, Z.; Yang, L.; HongHua, Y.; LiHua, L.; Feng, S.; LanYing, C., Quality evaluation of Dalbergiae Odoriferae Lignum by HPLC fingerprint and multi-component quantitative analysis. *China journal of Chinese materia medica* **2022,** 47, (4), 959-966.http://doi.org/10.19540/j.cnki.cjcmm.20210319.301.
3. Singh, A.; Bajpai, V.; Kumar, S.; Kumar, B.; Srivastava, M.; Rameshkumar, K. B., Comparative profiling of phenolic compounds from different plant parts of six Terminalia species by liquid chromatography–tandem mass spectrometry with chemometric analysis. *Industrial Crops & Products* **2016,** 87, 236-246.http://doi.org/10.1016/j.indcrop.2016.04.048.
4. Abdalaziz, M. N.; Ali, M. M.; Gahallah, M. D.; Garbi, M. I.; Kabbashi, A. S., Evaluation of fixed oil, seed extracts, of Carum carvi L. *International Journal*

of Computational and Theoretical Chemistry **2017,** 5, (1), 1-8.http://doi.org/10.11648/j.ijctc.20170501.11.

5. Jiang, J.S. Studies on the Chemical Constituents and Bioactivities of *Carthamus tinctorius* L. Ph.D. Thesis, Peking Union Medical College, Beijing, China, 2009.
6. Cortés-Rojas, D. F.; Souza, C. R. F. d.; Oliveira, W. P., Clove (Syzygium aromaticum): a precious spice. *Asian Pacific journal of tropical biomedicine* **2014,** 4, (2), 90-96.http://doi.org/10.1016/S2221-1691(14)60215-X.
7. Gupta, A.; Kumar, R.; Bhattacharyya, P.; Bishayee, A.; Pandey, A. K., Terminalia bellirica (Gaertn.) roxb. (Bahera) in health and disease: A systematic and comprehensive review. *Phytomedicine* **2020,** 77, 153278.http://doi.org/10.1016/j.phymed.2020.153278.
8. Chang, W. L.; Cheng, B. C., One new and nine known flavonoids from Choerospondias axillaries and their in vitro antitumor, anti-hypoxia and antibacterial activities. *Molecules* **2014,** 19, (12), 21363-77.http://doi.org/10.3390/molecules191221363.
9. Liu, R. X.; Li, L.; Wang, Q.; Wang, W.; Bi, K. S.; Guo, D. A., Simultaneous determination of nine flavonoids in Dalbergia odorifera by LC. *Chromatographia* **2005,** 61, (7-8), 409-413.http://doi.org/10.1365/s10337-005-0520-0.
10. Zhao, C. F.; Liu, Y. Q.; Cong, D. L.; Zhang, H.; Yu, J. J.; Jiang, Y., Screening and determination for potential α-glucosidase inhibitory constituents from Dalbergia odorifera T. Chen using ultrafiltration-LC/ESI-MS(n). *Biomedical chromatography : BMC* **2013,** 27, (12), 1621-9.http://doi.org/10.1002/bmc.2970.
11. Feng, J.; Yang, X.-W.; Wang, R.-F., Bio-assay guided isolation and identification of α-glucosidase inhibitors from the leaves of Aquilaria sinensis. *Phytochemistry* **2011,** 72, (2-3), 242-247.http://doi.org/10.1016/j.phytochem.2010.11.025.
12. Wang, S.-L.; Hwang, T.-L.; Chung, M.-I.; Sung, P.-J.; Shu, C.-W.; Cheng, M.-J.; Chen, J.-J., New flavones, a 2-(2-phenylethyl)-4 H-chromen-4-one derivative, and anti-inflammatory constituents from the stem barks of Aquilaria sinensis. *Molecules* **2015,** 20, (11), 20912-20925.http://doi.org/10.3390/molecules201119736.
13. Dan, C.; Jiao, W., Chemical Constituents of Caryophylli Flos. *Journal of Chinese Medicinal Materials* **2018,** 41, (05), 1108-1113.http://doi.org/10.13863/j.issn1001-4454.2018.05.020.
14. Shi, L. Study on the Chemical Constituents and In Vitro Anticancer Activity of Tibetan Medicine Terminalia chebula. Doctor, Beijing University of Chinese Medicine, 2020.
15. Fan, H. Q. Identification of phenolic compound in Amomum tsaoko Crevost et Lemaire and their hypoglycemic activity. Master, Nanchang University, 2023.
16. Yang, L. M.; Yang, L. J.; Jia, P.; Lan, W.; Zhang, Y. J.; Wang, S. X., HPLC-Q-TOF-MS/MS-based analysis of chemical constituents in Choerospondiatis fructus. *Academic Journal of Naval Medical University* **2016,** 37, (02), 159-166.http://doi.org/10.16781/j.0258-879x.2016.02.0159.

17. Li, F.; He, Z. S.; Ye, Y., Isocartormin, a novel quinochalcone C-glycoside from Carthamus tinctorius. *Acta Pharm Sin B* **2017,** 7, (4), 527-531.http://doi.org/10.1016/j.apsb.2017.04.005.
18. Sarabhai, S.; Sharma, P.; Capalash, N., Ellagic acid derivatives from Terminalia chebula Retz. downregulate the expression of quorum sensing genes to attenuate Pseudomonas aeruginosa PAO1 virulence. *PLoS ONE* **2017,** 8, (1), e53441.http://doi.org/10.1371/journal.pone.0053441.
19. Zhang, H.; Duan, C. P.; Luo, X.; Feng, Z. M.; Yang, Y. N.; Zhang, X., Two new quinochalcone glycosides from the safflower yellow pigments. *J Asian Nat Prod Res* **2020,** 22, (12), 1130-1137.http://doi.org/10.1080/10286020.2020.1846530.
20. Yue, S. J.; Tang, Y. P.; Xu, C. M.; Li, S. J.; Zhu, Y.; Duan, J. A., Two new quinochalcone C-glycosides from the florets of Carthamus tinctorius. *International Journal of Molecular Sciences* **2014,** 15, (9), 16760-71.http://doi.org/10.3390/ijms150916760.
21. Wu, J.; Hassan, S. S. U.; Zhang, X.; Li, T.; Rehman, A., Discovery of potent anti-MRSA components from Dalbergia odorifera through UPLC-Q-TOF-MS and targeting PBP2a protein through in-depth transcriptomic, in vitro, and in-silico studies. *J Pharm Anal* **2024,** 14, (8), 100938.http://doi.org/10.1016/j.jpha.2024.01.006.
22. Zhang, T. T.; Lu, C. L.; Jiang, J. G., Bioactivity evaluation of ingredients identified from the fruits of Amomum tsaoko Crevost et Lemaire, a Chinese spice. *Food & function* **2014,** 5, (8), 1747-54.http://doi.org/10.1039/c4fo00169a.
23. Yu, L. Q.; Shirai, N.; Suzuki, H.; Sugane, N.; Hosono, T.; Nakajima, Y.; Kajiwara, M.; Takatori, K., The effect of methanol extracts of tsao-ko (Amomum tsao-ko Crevost et Lemaire) on digestive enzyme and antioxidant activity in vitro, and plasma lipids and glucose and liver lipids in mice. *Journal of nutritional science and vitaminology* **2010,** 56, (3), 171-176.http://doi.org/10.3177/jnsv.56.171.
24. Zhang, K.; Chen, X. L.; Zhao, X.; Ni, J. Y.; Wang, H. L.; Han, M.; Zhang, Y. M., Antidiabetic potential of Catechu via assays for α-glucosidase, α-amylase, and glucose uptake in adipocytes. *Journal of Ethnopharmacology* **2022,** 291, 115118
25. Yu, W. Study on the Extraction and Isolation of Chemical Constituents from Carthamus tinctorius and Their Distribution in Rats. Master, Inner Mongolia University, 2019.
26. Jiang, J. S. Studies on the chemical constituents and bioactivities of Carthamus tinctorius L. Doctor Peking Union Medical College, 2009.
27. Li, X. C.; Wang, H. Q.; Liu, C.; Chen, R. Y., Chemical constituents of Acacia catechu. *China Journal of Chinese Materia Medica* **2010,** 35, (11), 1425-1427
28. Fan, H. Q.; Chen, M. S.; Dai, T. T.; Deng, L. Z.; Liu, C. M.; Zhou, W., Phenolic compounds profile of Amomum tsaoko Crevost et Lemaire and their antioxidant and hypoglycemic potential. *Food Bioscience* **2023,** 52.http://doi.org/10.1016/J.FBIO.2023.102508.
29. Wu, P. L.; Sun, L.; Qi, S. Y.; Tie Xin Zeng; Hou, Z. Y.; Yang, Y.; Xu, L. J.; Wei, J. H.; Xiao, P. G., Extraction process, chemical profile, and biological activity of aromatic oil from agarwood leaf (Aquilaria sinensis) by supercritical carbon dioxide extraction. *Journal of CO2 Utilization* **2023,** 77,

102615.http://doi.org/10.1016/J.JCOU.2023.102615.

30. Wang, M. R.; Li, W.; Luo, S.; Zhao, X.; Ma, C. H.; Liu, S. X., GC-MS Study of the Chemical Components of Different Aquilaria sinensis (Lour.) Gilgorgans and Agarwood from Different Asian Countries. *Molecules* **2018,** 23, (9), 2168.http://doi.org/10.3390/molecules23092168.

31. Yu, X. L.; Wang, W.; Yang, M., Antioxidant activities of compounds isolated from Dalbergia odorifera T. Chen and their inhibition effects on the decrease of glutathione level of rat lens induced by UV irradiation. *Food Chemistry* **2006,** 104, (2), 715-720.http://doi.org/10.1016/j.foodchem.2006.10.081.

32. Lee;, C.; Lee;, J. W.; Jin;, Q.; Jang;, D. S.; Lee;, S.-J.; Lee;, D., Inhibitory constituents of the heartwood of Dalbergia odorifera on nitric oxide production in RAW 264.7 macrophages. *Bioorg Med Chem Lett* **2013,** 23, (14), 4263-6.http://doi.org/10.1016/j.bmcl.2013.04.032.

33. Wang, S. H.; Cheng, J. T.; Guo, C.; Cui, W. J.; Shi, J.; Liu, A., Chemical constituents of Phyllanthus emblica and its anti-inflammation activities. *Chinese Traditional and Herbal Drugs* **2019,** 50, (20), 4873-4878

34. Shareena, S.; Vimal, N.; Luis, C., Protective Role of Phenolic Compounds from Whole Cardamom (Elettaria cardamomum (L.) Maton) against LPS-Induced Inflammation in Colon and Macrophage Cells. *Nutrients* **2023,** 15, (13), 2965.http://doi.org/10.3390/nu15132965.

35. Hu, Y. F.; Gao, X. Y.; Zhao, Y.; Liu, S. F.; Luo, K. L.; Fu, X.; Li, J. Y., Flavonoids in Amomum tsaoko Crevost et Lemarie Ameliorate Loperamide-Induced Constipation in Mice by Regulating Gut Microbiota and Related Metabolites. *Int J Mol Sci* **2023,** 24, (8).http://doi.org/10.3390/ijms24087191.

36. Kumar, S. A.; Laxman, S.; Sultan, Z.; Dhiraj, S.; Ranjan, M.; Kumar, R. R.; Sayeed, A., LC-MS/MS-based Targeted Metabolomic Profiling of Aqueous and Hydro-alcoholic Extracts of Pistacia integerrima Linn., Quercus infectoria Olivier and Terminalia chebula Retz. *Pharmacognosy Magazine* **2023,** 19, (2), 222-230.http://doi.org/10.1177/09731296221144809.

37. Li, G. Z.; Li, X. J.; Cao, L.; Zhang, L. J.; Shen, L. G.; Zhu, J.; Wang, J. C.; Si, J. Y., Chemical constituents from seeds of Ferula sinkiangensis. *Chinese Traditional and Herbal Drugs* **2015,** 46, (12), 1730-1736

38. Yang, B.; Kortesniemi, M.; Liu, P.; Karonen, M.; Salminen, J. P., Analysis of hydrolyzable tannins and other phenolic compounds in emblic leafflower (Phyllanthus emblica L.) fruits by high performance liquid chromatography-electrospray ionization mass spectrometry. *J Agric Food Chem* **2012,** 60, (35), 8672-83.http://doi.org/10.1021/jf302925v.

39. Omidpanah, S.; Vahedi-Mazdabadi, Y.; Manayi, A.; Rastegari, A.; Hariri, R.; Mortazavi-Ardestani, E.; Eftekhari, M.; Khanavi, M.; Akbarzadeh, T.; Saeedi, M., Phytochemical investigation and anticholinesterase activity of ethyl acetate fraction of Myristica fragrans Houtt. seeds. *Natural Product Research* **2022,** 36, (2), 610-616.http://doi.org/10.1080/14786419.2020.1788555.

40. Tursun, E. Study on the Chemical Constituents from Safflower and Acorn. Master, Tarim University, 2017.

41. Song, Z. B.; Chen, T.; Wang, S.; Shen, C.; Ma, Y. M.; Li, A. J.; Chen, Z.; Li, Y. L., Large-scale preparation of five polar polyphenols including three isomers

from Phyllanthus emblica Linn. by preparative high - speed counter - current chromatography. *Journal of Separation Science* **2023,** 46, (2), 2200748.http://doi.org/10.1002/jssc.202200748.

42. Mingkwan, N. T.; Ariyaphong, W.; Kanjana, J.; Noppamas, S.; Seewaboon, S., Antioxidant and Antitumorigenic Activitiesof the Standardized Water Extract From Fruit of Terminalia chebula Retz. var. chebula. *Natural Product Communications* **2023,** 18, (6).http://doi.org/10.1177/1934578X231176925.
43. Yang, X.; Shen, C.; Li, H. M.; Wang, N. N.; Ma, J. L., Combined chromatographic strategy based on macroporous resin, high‐speed counter‐current chromatography and preparative HPLC for systematic separation of seven antioxidants from the fruit of Terminalia billerica. *Journal of Separation Science* **2019,** 42, (20), 3191-3199
44. Li, N. N.; Li, S. Q.; Wang, Q. R.; Yang, S. Y.; Hou, Y. N.; Gao, Y., A novel visualization method for the composition analysis of processed garlic by MALDI-TOF imaging mass spectrometry (MSI) and Q-TOF LC-MS/MS. *Food Res Int* **2023,** 168, 112746.http://doi.org/10.1016/j.foodres.2023.112746.
45. Rajmohamed, M. A.; Natarajan, S.; Palanisamy, P.; Abdulkader, A. M.; Govindaraju, A., Antioxidant and Cholinesterase Inhibitory Activities of Ethyl Acetate Extract of Terminalia chebula: Cell-free In vitro and In silico Studies. *Pharmacogn Mag* **2017,** 13, (Suppl 3), S437-s445.http://doi.org/10.4103/pm.pm_57_17.
46. Singh, G.; Kumar, P., Extraction, gas chromatography–mass spectrometry analysis and screening of fruits of Terminalia chebula Retz. for its antimicrobial potential. *Pharmacognosy Research* **2013,** 5, (3), 162-168.http://doi.org/10.4103/0974-8490.112421.
47. Oliveira, M. S. d.; Costa, W. A. d.; Pereira, D. S.; Botelho, J. R. S.; Menezes, T. O. d. A.; Andrade, E. H. d. A.; Silva, S. H. M. d.; Filho, A. P. d. S. S.; Carvalho, R. N. d., Chemical composition and phytotoxic activity of clove (Syzygium aromaticum) essential oil obtained with supercritical CO_2. *The Journal of Supercritical Fluids* **2016,** 118, 185-193.http://doi.org/10.1016/j.supflu.2016.08.010.
48. Divya, K.; Ramalakshmi, K.; Murthy, P. S.; Rao, L. J. M., Volatile oils from Ferula asafoetida varieties and their antimicrobial activity. *LWT - Food Science and Technology* **2014,** 59, (2), 774-779.http://doi.org/10.1016/j.lwt.2014.07.013.
49. Ma, R.; Liu, H.; Shi, F.; Fu, Y.; Wei, P.; Liu, Z., The chemical composition and antioxidant activity of essential oils and extracts of Dalbergia odorifera leaves. *Holzforschung* **2020,** 74, (8), 755-763.http://doi.org/doi:10.1515/hf-2019-0155.
50. Zhang, X. H.; Silva, J. A. T. d.; Jia, Y. X.; Zhao, J. T.; Ma, G. H., Chemical composition of volatile oils from the pericarps of Indian sandalwood (Santalum album) by different extraction methods. *Nat Prod Commun* **2012,** 7, (1), 93-6.http://doi.org/10.1177/1934578X1200700132.
51. Elgersma, A.; Søegaard, K.; Jensen, S. K., Fatty acids, α-tocopherol, β-carotene, and lutein contents in forage legumes, forbs, and a grass-clover mixture. *J Agric Food Chem* **2013,** 61, (49), 11913-20.http://doi.org/10.1021/jf403195v.
52. Ren, H. T.; Lian, Q.; Zhou, H. C.; Wu, L. Z.; Fan, Y. H., Analysis and Evaluation of Fatty Acid Composition in Amomum tsao-ko Seed Oil in Different

Producing Areas of Yunnan. *Tropical Agricultural Science & Technology* **2023,** 46, (01), 29-34.http://doi.org/10.16005/j.cnki.tast.2023.01.007.

53. Mouming, Z.; Xiaoli, L.; Chun, C.; Wei, L., Composition and Antimicrobial Activity of Essential Oil from Phyllanthus emblica L. by Supercritical CO2Extraction. *Journal of South China University of Technology(Natural Science Edition)* **2007**, (12), 116-120
54. Balkrishna, A.; Maheswari, P. R.; Joshi, M.; Varshney, Y.; Jangid, H.; Kabdwal, M.; Lochab, S.; Nain, P.; Varshney, A., In-depth chromatographic and spectrally defined phytocompounds in super-critical-CO2 extracted Phyllanthus emblica seeds oil show robust anti-microbial/biofilm effects in the non-mutagenic manner. *Applied Food Research* **2025,** 5, (2), 101116-101116.http://doi.org/10.1016/J.AFRES.2025.101116.
55. Wong, X. K.; Alasalvar, C.; Bo, S.; Pan, J.; Chang, S. K., Unlocking the power of nutmeg: Nutritional composition, phytochemicals, and health benefits revealed through chemometrics and multi-omics advances. *Food Res Int* **2025,** 218, 116798.http://doi.org/10.1016/j.foodres.2025.116798.
56. Song, W. F.; Liao, M. J.; Luo, S. Y., Analyze on chemical compositions of Dalbergia odorifera essential oils extracted by CO2-supercritical-fluid-extraction and steam distillation extraction. *Journal of Chinese medicinal materials* **2011,** 34, (11), 1725-7
57. Li, H. M.; Qu, Y. F.; Zhang, J. W.; Zhang, J. Z., Spasmolytic activity of Aquilariae Lignum Resinatum extract on gastrointestinal motility involves muscarinic receptors, calcium channels and NO release. *Pharmaceutical biology* **2018,** 56, (1), 559-566.http://doi.org/10.1080/13880209.2018.1492000.
58. Bai, X.; Zeng, Q. Q.; Ma, J. L.; Sun, L. Q.; Lu, Y. C., Chemical composition and antibacterial activity analysis of Carum carvi L. by supercritical CO2extraction technique. *China Food Additives* **2016**, (02), 106-111
59. Peng, D.-Q.; Yu, Z.-X.; Wang, C.-H.; Gong, B.; Liu, Y.-Y.; Wei, J.-H., Chemical constituents and anti-inflammatory effect of incense smoke from agarwood determined by GC-MS. *International Journal of Analytical Chemistry* **2020,** 2020, 1-19.http://doi.org/10.1155/2020/4575030.
60. Qu, C.; YUE, S. J.; Lin, H.; Kai, J.; Shang, G. X.; Tang, Y. P., Chemical constituents of Carthamus tinctorius. *Chinese Traditional and Herbal Drugs* **2015,** 46, (13), 1872-1877
61. Pfundstein, B.; El Desouky, S. K.; Hull, W. E.; Haubner, R.; Erben, G.; Owen, R. W., Polyphenolic compounds in the fruits of Egyptian medicinal plants (Terminalia bellerica, Terminalia chebula and Terminalia horrida): characterization, quantitation and determination of antioxidant capacities. *Phytochemistry* **2010,** 71, (10), 1132-48.http://doi.org/10.1016/j.phytochem.2010.03.018.
62. Lee, D. Y.; Kim, H. W.; Yang, H.; Sung, S. H., Hydrolyzable tannins from the fruits of Terminalia chebula Retz and their α-glucosidase inhibitory activities. *Phytochemistry* **2017,** 137, 109-116.http://doi.org/10.1016/j.phytochem.2017.02.006.
63. Li, Y. J.; Liang, C. C.; Jin, L.; Chen, J., Inhibition mechanisms of four ellagitannins from terminalia chebula fruits on acetylcholinesterase by inhibition kinetics, spectroscopy and molecular docking analyses. *Spectrochimica acta. Part A, Molecular and biomolecular spectroscopy* **2023,** 302, 123115-123115.http://doi.org/10.1016/J.SAA.2023.123115.

64. Yan, X. Y.; Li, Q. J.; Jing, L.; Wu, S. Y.; Duan, W.; Chen, Y.; Chen, D. Y.; Pan, X. Q., Current advances on the phytochemical composition, pharmacologic effects, toxicology, and product development of Phyllanthi Fructus. *Frontiers in Pharmacology* **2022,** 13, 1017268-1017268.http://doi.org/10.3389/FPHAR.2022.1017268.
65. J, P. F.; S, W.; M, T. M.; E, P. S.; S, T.; B, K. J.; S, T. D. A.; V, P. J.; J, L. R., GC-MS method for identification of organic chemical compounds nutmeg flesh of North Minahasa local varieties. *IOP Conference Series: Earth and Environmental Science* **2023,** 1241, (1).http://doi.org/10.1088/1755-1315/1241/1/012004.
66. Pillai, P. S.; Roziahanim, M.; Shah, A. M. A. M.; Sabariah, I.; Nin, M. C., Chemical composition, antioxidant and cytotoxicity activities of the essential oils of Myristica fragrans and Morinda citrifolia. *Journal of the science of food and agriculture* **2012,** 92, (3), 593-7.http://doi.org/10.1002/jsfa.4613
67. Liu, F. S.; Zhang, T. T.; Xu, J.; Jing, Q. X.; Gong, C., New tirucallane-type triterpenoids from the resin of Boswellia carteriiand their NO inhibitory activities. *Chin J Nat Med* **2021,** 19, (9), 686-692.http://doi.org/10.1016/s1875-5364(21)60099-7.
68. Tiwana, G.; Cock, I. E.; White, A.; Cheesman, M. J., Use of specific combinations of the triphala plant component extracts to potentiate the inhibition of gastrointestinal bacterial growth. *J Ethnopharmacol* **2020,** 260, 112937.http://doi.org/10.1016/j.jep.2020.112937.
69. Mushtaq, S.; Aga, M. A.; Qazi, P. H.; Ali, M. N.; Shah, A. M.; Lone, S. A.; Shah, A.; Hussain, A.; Rasool, F.; Dar, H.; Shah, Z. H.; Lone, S. H., Isolation, characterization and HPLC quantification of compounds from Aquilegia fragrans Benth: Their in vitro antibacterial activities against bovine mastitis pathogens. *J Ethnopharmacol* **2016,** 178, 9-12.http://doi.org/10.1016/j.jep.2015.11.039.
70. Zhao, Y. Y.; Xi, C.; Liu, D. H.; Ren, X. Q.; Fan, J. Y.; Tangthianchaichana, J.; Lu, Y.; Wu, H., Chemical components with antibacterial properties found in sanchen powder from traditional Tibetan medicine. *Journal of ethnopharmacology* **2024,** 326, 117981.http://doi.org/10.1016/J.JEP.2024.117981.
71. Li, F.; Yang, W.; Yang, M.; Wang, Y.; Zhang, J., Differences between two plants fruits: Amomum tsaoko and Amomum maximum, using the SPME-GC–MS and FT-NIR to classification. *Arabian Journal of Chemistry* **2024**, 105665
72. Thi, O. V.; Viet, P. N.; Sun, M. B.; Young, Y. S.; Ah, K. J., Insights into the inhibitory activities of neolignans and diarylnonanoid derivatives from nutmeg (Myristica fragrans Houtt.) seeds on soluble epoxide hydrolase using in vitro and in silico approaches. *Journal of enzyme inhibition and medicinal chemistry* **2023,** 38, (1), 2251099-2251099.http://doi.org/10.1080/14756366.2023.2251099.
73. Liu, W. X.; Cheng, X. L.; Guo, X. H.; Hu, X. R.; Wei, F.; Ma, S. C., Identification of Calculus Bovis and its mixed varieties by ultra-high-performance liquid chromatography/quadrupole time-of-flight mass spectrometry (UHPLC-Q/TOF-MS) combined with the principal component analysis (PCA) method. *J Pharm Biomed Anal* **2020,** 179, 112979.http://doi.org/10.1016/j.jpba.2019.112979.
74. Wolosik, K.; Markowska, A., Amaranthus Cruentus Taxonomy, Botanical Description, and Review of its Seed Chemical Composition. *Natural Product Communications* **2019,** 14, (5), 1934578X19844141-1934578X19844141.http://doi.org/10.1177/1934578X19844141.

75. Gao, H.; Huang, Y. N.; Gao, B.; KAWABATA, J., Chebulagic Acid Is a Potent α-Glucosidase Inhibitor. *Bioscience, Biotechnology, and Biochemistry* **2008,** 72, (2), 601-603.http://doi.org/10.1271/bbb.70591.
76. Hamm, S.; Bleton, J.; Connan, J.; Tchapla, A., A chemical investigation by headspace SPME and GC-MS of volatile and semi-volatile terpenes in various olibanum samples. *Phytochemistry* **2005,** 66, (12), 1499-514.http://doi.org/10.1016/j.phytochem.2005.04.025.
77. Misra, B. B.; Das, S. S.; Dey, S., Volatile profiling from heartwood of East Indian sandalwood tree. *Journal of Pharmacy Research* **2013,** 7, (4), 299-303.http://doi.org/10.1016/j.jopr.2013.04.030.
78. Tu, Y. Y.; Huang, Y. X.; Qiu, F., Analysis of the Components of Santalum album and Its Incense Smoke by Thermal Desorption-GC/MS. *Chinese Traditional Patent Medicine* **2020,** 42, (01), 243-247
79. Vesna, N.; Ljubisa, N.; Ana, D.; Ivana, G.; Maja, U.; Ljiljana, S.; Jelena, S.; Bojana, D., Chemical Composition, Antioxidant and Antimicrobial Activity of Nutmeg (;Houtt.) Seed Essential Oil. *Journal of Essential Oil Bearing Plants* **2021,** 24, (2), 218-227.http://doi.org/10.1080/0972060X.2021.1907230.
80. Zhang, X.; Wang, L. X.; Hao, R.; Huang, J. J.; Zargar, M., Sesquiterpenoids in Agarwood: Biosynthesis, Microbial Induction, and Pharmacological Activities. *J Agric Food Chem* **2024,** 72, (42), 23039-23052.http://doi.org/10.1021/acs.jafc.4c06383.
81. Liao, L. K.; Yang, S. T.; Li, R. Y.; Zhou, W.; Xiao, Y.; Yuan, Y.; Cha, Y. S.; He, G. F.; Li, J. H., Anti-inflammatory effect of essential oil from Amomum Tsaoko Crevost et Lemarie. *Journal of Functional Foods* **2022,** 93.http://doi.org/10.1016/J.JFF.2022.105087.
82. Braun, N. A.; Sim, S.; Kohlenberg, B.; Lawrence, B. M., Hawaiian sandalwood: oil composition of Santalum paniculatum and comparison with other sandal species. *Nat Prod Commun* **2014,** 9, (9), 1365-8.http://doi.org/10.1177/1934578X1400900936.
83. Rokas, M.; Gintarė, J.; Vytautas, R.; Valeryia, M.; Inga, M.; Mindaugas, M.; Kęstutis, M.; Jurga, B.; Arvydas, S. V., The effect of nutmeg essential oil constituents on Novikoff hepatoma cell viability and communication through Cx43 gap junctions. *Biomedicine & Pharmacotherapy* **2021,** 135, 111229-111229.http://doi.org/10.1016/J.BIOPHA.2021.111229.
84. Cai, J. L.; Chen, P. P.; Lan, B. M.; Lin, T., Study on extraction,component analysis and bacteriostasis effect of garlic essential oil. *Jiangsu Agricultural Sciences* **2020,** 48, (23), 186-190.http://doi.org/10.15889/j.issn.1002-1302.2020.23.038.
85. Okiki, P. A.; Nwobi, C. P.; Akpor, O. B.; Adewole, E.; Agbana, R. D., Assessment of nutritional and medicinal properties of nutmeg. *Scientific African* **2023,** 19.http://doi.org/10.1016/J.SCIAF.2023.E01548.
86. Zhang, T. T.; Lu, C. L.; Jiang, J. G., Antioxidant and anti-tumour evaluation of compounds identified from fruit of Amomum tsaoko Crevost et Lemaire. *Journal of Functional Foods* **2015,** 18, 423-431.http://doi.org/10.1016/j.jff.2015.08.005.
87. Chang, Z. R.; Xue, Q.; He, M. Y.; Liu, C. Y.; Gao, P., Research Progress on Carum carvi and Prediction of Its Quality Markers. *Journal of Chinese Medicinal*

Materials **2023**, (10), 2620-2627.http://doi.org/10.13863/j.issn1001-4454.2023.10.041.

88. PAN, C.; JU, X. R.; XU, Z. J.; Yuan, J., Study on the Extraction Technology of Garlic Oil with Ethanol Solvent and Analysis of Aroma Components by GC-MS. *China Condiment* **2017,** 42, (02), 19-23
89. Mohankumar, A.; Kalaiselvi, D.; Levenson, C.; Shanmugam, G.; Thiruppathi, G.; Nivitha, S.; Sundararaj, P., Antioxidant and stress modulatory efficacy of essential oil extracted from plantation-grown Santalum album L. *Industrial Crops & Products* **2019,** 140, 111623-111623.http://doi.org/10.1016/j.indcrop.2019.111623.
90. Liu, Y. L.; Wang, M. N.; Cao, Y. G.; Zeng, M. N.; Zhang, Q. Q.; Ren, Y. J., Chemical Constituents from the Flowers of Carthamus tinctorius L. and Their Lung Protective Activity. *Molecules* **2022,** 27, (11).http://doi.org/10.3390/molecules27113573.
91. Matsuo, Y.; Mimaki, Y., α-Santalol derivatives from Santalum album and their cytotoxic activities. *Phytochemistry* **2012,** 77, 304-11.http://doi.org/10.1016/j.phytochem.2012.02.007.
92. Sciarrone, D.; Costa, R.; Ragonese, C.; Tranchida, P. Q.; Tedone, L.; Santi, L.; Dugo, P.; Dugo, G.; Mondello, L., Application of a multidimensional gas chromatography system with simultaneous mass spectrometric and flame ionization detection to the analysis of sandalwood oil. *J Chromatogr A* **2011,** 1218, (1), 137-42.http://doi.org/10.1016/j.chroma.2010.10.117.
93. Zhao, X. B.; Mei, W. L.; Gong, M. F.; Bai, H. J.; Dai, H. F., Study on chemical compositions and antibacterial activity of the volatile oil from Dalbergiae odoriferae. *Guangdong Agricultural Sciences* **2012,** 39, (03), 95-99.http://doi.org/10.16768/j.issn.1004-874x.2012.03.020.
94. Hadidi, M.; Pouramin, S.; Adinepour, F.; Haghani, S.; Jafari, S. M., Chitosan nanoparticles loaded with clove essential oil: Characterization, antioxidant and antibacterial activities. *Carbohydr Polym* **2020,** 236, 116075.http://doi.org/10.1016/j.carbpol.2020.116075.
95. Thi, V. Y.; Dien, D.; Minh, T. D.; Nam, T. T.; Hoi, N.; Thanh, D. T.; Quoc, D. T.; Thi, N. H.; A, O. I., The antimicrobial activity and essential oil constituents of the leaves and trunks of Aquilaria banaensis P.H.Hô (Thymelaeceae) from Vietnam. *Natural product research* **2023,** 38, (5), 1-9.http://doi.org/10.1080/14786419.2023.2196624.
96. Wang, J. L.; Meng, X. H.; Zheng, Y. D.; Sang, C. Y.; Wang, W. F.; Ma, J. Y., (±)-Ferulasin, unusual sesquiterpene chromones from Ferula sinkiangensis. *Tetrahedron* **2022,** 122, 132953.http://doi.org/10.1016/J.TET.2022.132953.
97. RongYe, W.; Hui, X.; YongXiang, W.; Hao, H.; BoKai, W.; Meng, D.; YueLin, S.; YunFang, Z.; Jiao, Z.; HuiXia, H.; Jun, L., Chemical constituents of diterpenoids from Boswellia carterii. *China journal of Chinese materia medica* **2023,** 48, (9), 2464-2470.http://doi.org/10.19540/j.cnki.cjcmm.20230202.201.
98. Kim, T. H.; Ito, H.; Hayashi, K.; Hasegawa, T.; Machiguchi, T.; Yoshida, T., Aromatic constituents from the heartwood of Santalum album L. *Chem Pharm Bull (Tokyo)* **2005,** 53, (6), 641-4.http://doi.org/10.1248/cpb.53.641.

99. li, X. D.; Luo, X. M.; Ma, Z.; Wu, Y.; Gong, P. Y., Research progress on chemical constructions and pharmacological effects of Terminaliae Belliricae Fructus and predictive analysis of its quality markers. *Chinese Traditional and Herbal Drugs* **2023,** 54, (03), 976-990

100. Sun, H. F.; Lv, Q.; Ji, X.; Fang, C.; Fei, J. X.; Liu, X. J.; Liu, J. X.; Liu, X. H., Three New Antioxidative Phenolics From Phyllanthus emblica L. Fruit. *Natural Product Communications* **2023,** 18, (3), 1934578X231155717.http://doi.org/10.1177/1934578X231155717.
